# Novel Secretion Apparatus Maintains Spore Integrity and Developmental Gene Expression in *Bacillus subtilis*

**DOI:** 10.1371/journal.pgen.1000566

**Published:** 2009-07-17

**Authors:** Thierry Doan, Cecile Morlot, Jeffrey Meisner, Monica Serrano, Adriano O. Henriques, Charles P. Moran, David Z. Rudner

**Affiliations:** 1 Department of Microbiology and Molecular Genetics, Harvard Medical School, Boston, Massachusetts, United States of America; 2 Department of Microbiology and Immunology, Emory University School of Medicine, Atlanta, Georgia, United States of America; 3 Microbial Development Group, Instituto de Tecnologia Química e Biológica, Universidade Nova de Lisboa, Oeiras, Portugal; The University of North Carolina at Chapel Hill, United States of America

## Abstract

Sporulation in *Bacillus subtilis* involves two cells that follow separate but coordinately regulated developmental programs. Late in sporulation, the developing spore (the forespore) resides within a mother cell. The regulation of the forespore transcription factor σ^G^ that acts at this stage has remained enigmatic. σ^G^ activity requires eight mother-cell proteins encoded in the *spoIIIA* operon and the forespore protein SpoIIQ. Several of the SpoIIIA proteins share similarity with components of specialized secretion systems. One of them resembles a secretion ATPase and we demonstrate that the ATPase motifs are required for σ^G^ activity. We further show that the SpoIIIA proteins and SpoIIQ reside in a multimeric complex that spans the two membranes surrounding the forespore. Finally, we have discovered that these proteins are all required to maintain forespore integrity. In their absence, the forespore develops large invaginations and collapses. Importantly, maintenance of forespore integrity does not require σ^G^. These results support a model in which the SpoIIIA-SpoIIQ proteins form a novel secretion apparatus that allows the mother cell to nurture the forespore, thereby maintaining forespore physiology and σ^G^ activity during spore maturation.

## Author Summary

During development, cell–cell signaling pathways coordinate programs of gene expression in neighboring cells. Cell–cell signaling is typically achieved by the secretion of a signaling ligand followed by its binding to a membrane receptor on the surface of a neighboring cell (or cells). The ligand-bound receptor directly or indirectly triggers transcription factor activation. Here we present evidence for a non-canonical signaling pathway that links developmental gene expression in the forespore and mother cell during spore formation in *Bacillus subtilis*. Our data support a model in which a novel secretion system is assembled in the double membrane that encases the spore within the mother cell. This apparatus is not involved in transducing a specific activating signal but rather allows the mother cell to nurture the spore and thereby maintain the spore program of developmental gene expression.

## Introduction

How cells communicate with each other is a fundamental biological question relevant to the development of multi-cellular organisms, microbial pathogenesis and communities of microorganisms. In the bacterium *Bacillus subtilis*, cell-cell signaling pathways play a key role in coordinating gene expression during the process of spore formation. Upon the initiation of sporulation, the developing cell divides asymmetrically, generating two cells of unequal size and dissimilar fate: a small cell (the prospective spore), referred to as the forespore, and a large cell called the mother cell [1],[2]. Initially, these two cells lie side by side, but shortly after polar division the mother cell membranes migrate around the forespore in a phagocytic-like process, generating a cell within a cell. As a result of this engulfment process, the forespore is surrounded by two membranes, one derived from the mother cell, the other from the forespore. Shortly after the completion of engulfment, the mother cell packages the forespore in a protective coat while the forespore prepares for dormancy. Once the spore is fully mature, the mother cell lyses releasing it into the environment.

During this developmental process, the mother cell and forespore follow separate programs of gene expression directed by alternative sigma factors. However, cell-cell signaling pathways ensure that gene expression in one cell is coordinated with gene expression in the other [2]–[4]. Shortly after polar division, the first cell-type specific transcription factor (σ^F^) is activated in the forespore. σ^F^ is then responsible for the activation of σ^E^ in the mother cell. At a later stage, σ^E^ is required for the activation of σ^G^ in the forespore. Finally, σ^G^ sets in motion the activation of σ^K^ in the mother cell. The signal transduction pathways that govern the activation of the mother-cell transcription factors (σ^E^ and σ^K^) are well established [1],[2],[5]. By contrast, the mechanism underlying activation of σ^G^ in the forespore has remained enigmatic.

The late-acting forespore transcription factor σ^G^ appears to be regulated at multiple levels. First, the transcription of the gene encoding σ^G^ (*sigG*) is under the control of the earlier-acting forespore transcription factor σ^F^ [6],[7]. However, unlike the other σ^F^-dependent genes, *sigG* transcription is delayed by approximately 30 min by an as yet unknown mechanism [8]–[10]. Once synthesized, σ^G^ activity requires eight mother cell proteins encoded in the *spoIIIA* operon [11] and a forespore membrane protein SpoIIQ [12]. In addition, σ^G^ activity requires proper engulfment. Mutants that block the engulfment process are impaired in σ^G^-dependent gene expression [13]–[15]. Finally, once activated, σ^G^ recognizes its own promoter and its level increases rapidly in the forespore [6],[7].

It is not known how the SpoIIIA proteins in the mother cell and SpoIIQ in the forespore participate in regulating σ^G^. It has been suggested that they function to transduce a signal from the mother cell to trigger its activation [2], [4], [11], [16]–[18]. It has also been proposed that these proteins are involved in monitoring the process of engulfment and sending an activating signal to the forespore upon its completion.

Fluorescence microscopy experiments have revealed that SpoIIQ and SpoIIIAH (the last gene in the *spoIIIA* operon) both localize to the membranes that surround the forespore [19],[20]. Moreover, these two membrane proteins can interact across the double membrane [16],[20]. The relevance of this interaction for σ^G^ activation has been unclear. One clue to the role of these proteins in σ^G^ activation is that several of the SpoIIIA proteins share weak similarity with components of specialized secretion systems [16],[21],[22]. In addition, recent experiments suggest that SpoIIQ forms large pores in the forespore membrane [22]. Based on these observations, it has been proposed that the SpoIIIA proteins and SpoIIQ form a channel between the mother cell and forespore [21],[22]. This apparatus could transduce an activating signal to trigger σ^G^ activation. Camp and Losick have recently found that the activity of transcription factors other than σ^G^ also require the SpoIIIA proteins and SpoIIQ [23]. Based on these findings, they have proposed that this putative channel serves as a “feeding tube” allowing the mother cell to nurture the forespore by providing small molecules needed for biosynthetic activity. In this model, these molecules are necessary to feed the forespore rather than as a signal to activate σ^G^.

Here we show that SpoIIIAA shares strong similarity to ATPases of the type II and IV secretion systems and that the conserved ATPase motifs are required for σ^G^ activity and spore-formation. Moreover, we demonstrate that at least six of the SpoIIIA proteins in the mother cell and SpoIIQ in the forespore are present in a multimeric membrane complex that spans the double membrane surrounding the forespore. Finally, we show that SpoIIQ, the SpoIIIA proteins and the ATPase motifs in SpoIIIAA are all required to maintain forespore integrity. In their absence, the forespore develops large invaginations and appears to collapse. Importantly, maintenance of forespore integrity does not require σ^G^ indicating that forespore collapse is not a result of the failure to activate σ^G^ (and the late program of forespore gene expression under its control). Instead, these results suggest that the block to σ^G^ activity in the SpoIIIA and SpoIIQ mutants is a manifestation of the failure to maintain forespore integrity. Consistent with this idea, we demonstrate that premature synthesis of σ^G^ results in early σ^G^ activity that does not require SpoIIIA or SpoIIQ whereas late σ^G^ activity requires these proteins. Collectively, these data support and extend the feeding-tube model of Camp and Losick in which SpoIIIA-SpoIIQ form a novel secretion apparatus that allows the mother cell to nurture the forespore at late stages of development.

## Results

### The conserved ATPase motifs in SpoIIIAA are required for σ^G^ activation

It has been reported previously that the first gene in the *spoIIIA* operon (*spoIIIAA*) is homologous to secretion superfamily ATPases [16],[22],[24]. Sequence identity searches indicate that SpoIIIAA most closely resembles proteins in the family of NTPases involved in type II and type IV secretion (Figure S1). All members of this family contain four highly conserved motifs: the Walker A and B boxes involved in nucleotide-binding and hydrolysis and two additional motifs called the Aspartate and Histidine boxes. The Aspartate and Histidine residues in these two motifs are present in close proximity to the nucleotide binding pocket and are thought to participate in NTP-binding or hydrolysis [25].

**Figure 1:**
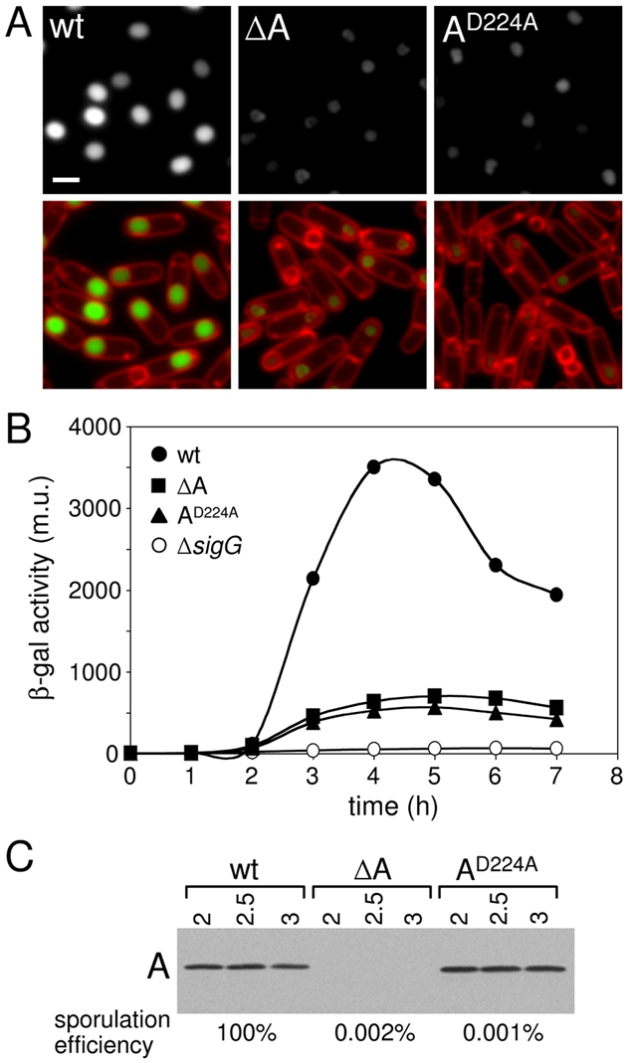
The ATPase motifs in SpoIIIAA are required for σ^G^ activity and sporulation efficiency. (A) σ^G^ activity was assessed in single cells by microscopy using a fluorescent reporter (P*_sspE_-cfp*) in a wild-type background (wt, BTD2779), a Δ*spoIIIAA* mutant (ΔA, BTD2713) and a *spoIIIAA* ^D224A^ mutant (A^D224A^, BTD2775). Cells were visualized at hour 3 of sporulation. Forespore CFP fluorescence (false-colored green in the lower panel) and the fluorescent membrane dye TMA-DPH (false-colored red) are shown. Scale bar, 1 µm. (B) Expression of a σ^G^-dependent *sspB-lacZ* translational fusion [53] was monitored during a time course of sporulation in a wild-type background (wt, BTD2919), a Δ*spoIIIAA* mutant (ΔA, BTD2917), a *spoIIIAA* ^D224A^ mutant (A^D224A^, BTD2920) and a strain lacking σ^G^ (ΔsigG, BTD1331). Samples from sporulating cells were taken every hour and β-galactosidase activity (Miller Unit, m.u.) was determined. (C) Immunoblot analysis of whole cell lysates from sporulating cells shown in A using anti-SpoIIIAA antibodies. Time (in hours) after the initiation of sporulation is indicated. Sporulation efficiencies of the same strains are shown below the immunoblot.

To investigate whether SpoIIIAA is an ATPase and whether this activity is important for σ^G^ activation, we made amino acid substitutions in highly conserved residues in the four conserved motifs (K149A in the Walker A box, D224A in the Walker B box, E180Q in the Aspartate box, and H250Y in the Histidine box). Similar mutations in other ATPases have been shown to abrogate ATP binding or hydrolysis [26]–[28]. The *spoIIIAA* mutants were introduced into a *B. subtilis* strain harboring an in-frame deletion in the *spoIIIAA* gene and tested for their ability to activate σ^G^ during sporulation. σ^G^ activity was assessed in single cells by fluorescence microscopy using a σ^G^-responsive promoter (P*_sspE_*) fused to *cfp* and in a population-based assay using a σ^G^-responsive promoter (P*_sspB_*) fused to *lacZ*. Mutations in all four of the conserved motifs blocked σ^G^ activation to the same extent as the *spoIIIAA* in-frame deletion (Figure 1A and 1B, Figure S2). Moreover, the sporulation efficiency of the mutants was similar to the *spoIIIAA* null (Figure 1C and Figure S2). Importantly, almost all of the SpoIIIAA mutant proteins were produced at levels similar to wild-type SpoIIIAA (Figure 1C and Figure S2). The NTPases involved in type II and type IV secretion have been particularly refractory to biochemical reconstitution and our attempts to reconstitute SpoIIIAA ATPase activity *in vitro* were unsuccessful. Based on the mutational analysis, we tentatively conclude that SpoIIIAA is an ATPase and that ATPase activity is necessary for σ^G^ activation and efficient sporulation.

### The SpoIIIA proteins form a multimeric complex in the membranes that surround the forespore

**Figure 2:**
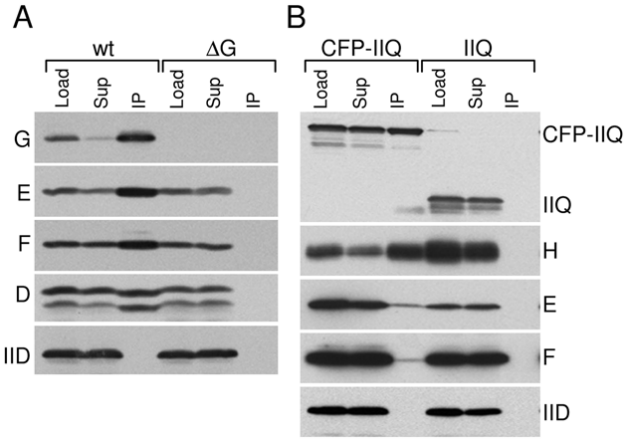
The SpoIIIA proteins and SpoIIQ reside in a membrane complex. Immunoprecipitations were performed on detergent-solubilized membrane fractions derived from *B. subtilis* sporulating cells at hour 2.5 of sporulation. Lysates were normalized based on the optical density of the cell cultures. (A) Immunoprecipitates using anti-SpoIIIAG antibody resin from a *spoIIIAG*+ strain (wt, BTD2869) and a Δ*spoIIIAG* mutant (ΔG, BDT2867) are shown. Both strains contain a myc-tagged *spoIIIAD* gene inserted at a non-essential locus. The detergent-solubilized membrane fraction prior to immunoprecipitation (Load), the supernatants after immunoprecipitation (Sup), and the immunoprecipitates (IP) were subjected to immunoblot analysis using anti-SpoIIIAG (G), anti-SpoIIIAE (E), anti-SpoIIIAF (F), anti-myc (D), and anti-SpoIID (IID) antibodies. (B) Immunoprecipitates using anti-GFP antibody resin from a Δ*spoIIQ* mutant harboring and a *cfp-spoIIQ* fusion at a non-essential locus (CFP-IIQ, BTD665), and a *spoIIQ*+ strain (IIQ, BDR94) are shown. The Load (L), Supernatant (Sup), and IP were subjected to immunoblot analysis using anti-GFP, anti-SpoIIIAH (H), anti-SpoIIIAF (F), anti-SpoIIIAE (E), and anti-SpoIID (IID). All four strains contained a Δ*spoIVB* mutation to prevent cleavage of proteins that have domains that reside in the intermembrane space (K. Marquis, N. Campo, T.D., and D.Z.R., unpublished observations).

Seven of the SpoIIIA proteins (SpoIIIAB through SpoIIIAH) are predicted to be integral membrane proteins and several share weak similarity to components of secretion complexes [21],[22] (see Discussion). To investigate whether the SpoIIIA proteins form a membrane complex, we performed co-immunoprecipitation experiments. Membrane extracts were prepared from sporulating cells collected at hour 2.5 of sporulation (see Material and Methods). The membrane proteins were then solubilized using the non-ionic detergent dodecyl maltoside (DDM) and SpoIIIAG was immunoprecipitated using anti-SpoIIIAG antibody resin. Consistent with the idea that the SpoIIIA proteins form a complex, SpoIIIAE, SpoIIIAF and a functional myc-tagged SpoIIIAD fusion were co-immunoprecipitated with SpoIIIAG (Figure 2A). Analysis of the immunoprecipitate by Mass Spectrometry also identified SpoIIIAB, for which we do not have an antibody. Importantly, a mother cell membrane protein (SpoIID) required for engulfment that localizes to the membranes surrounding the forespore [29] was not present in the immunoprecipitate (Figure 2A). Moreover, none of the SpoIIIA proteins were found in the immunoprecipitate from an extract derived from a strain lacking SpoIIIAG (Figure 2A). Similar results were obtained when immunoprecipitations were performed using anti-SpoIIIAF, anti-SpoIIIAE, or anti-Myc antibody resins (Figure S3 and data not shown). We note that in all of these experiments we were unable to detect SpoIIIAA in the immunoprecipitates (data not shown). Since detergent is required to solubilize the SpoIIIA proteins from the membrane, it is difficult to interpret the failure to detect a protein in the complex. All together, these results are consistent with the idea that SpoIIIAG, SpoIIIAE, SpoIIIAF, SpoIIIAD and SpoIIIAB are present in a membrane complex.

We and others have previously reported that SpoIIIAH and the forespore protein SpoIIQ can be efficiently co-immunoprecipitated from detergent-solubilized membrane extracts [16],[30]. Surprisingly, neither protein was detected in our immunoprecipitates (data not shown). To directly test whether or not the SpoIIIAH-SpoIIQ complex interacts with the other SpoIIIA proteins, we used a strain harboring a functional CFP-SpoIIQ fusion [20] and performed immunoprecipitations with anti-GFP antibody resin from membrane extracts solubilized with the mild detergent digitonin. Under these conditions, we were able to detect a small amount of SpoIIIAG, SpoIIIAE and SpoIIIAF in the immunoprecipitate (Figure 2B and data not shown). Importantly, none of the SpoIIIA proteins were found in the immunoprecipitate from an extract derived from a strain containing untagged SpoIIQ. Apparently, the larger SpoIIIA-SpoIIQ complex is sensitive to detergent. Consistent with this idea, SpoIIIAG and SpoIIIAH (but not an unrelated membrane protein) could easily be co-immunoprecipitated from a membrane fraction lacking detergent (Figure S4). All together, these results are consistent with the idea that a large complex composed of the SpoIIIA proteins and SpoIIQ is present in the two membranes that surround the forespore.

### SpoIIIAG localizes to the membranes that surround the forespore

**Figure 3:**
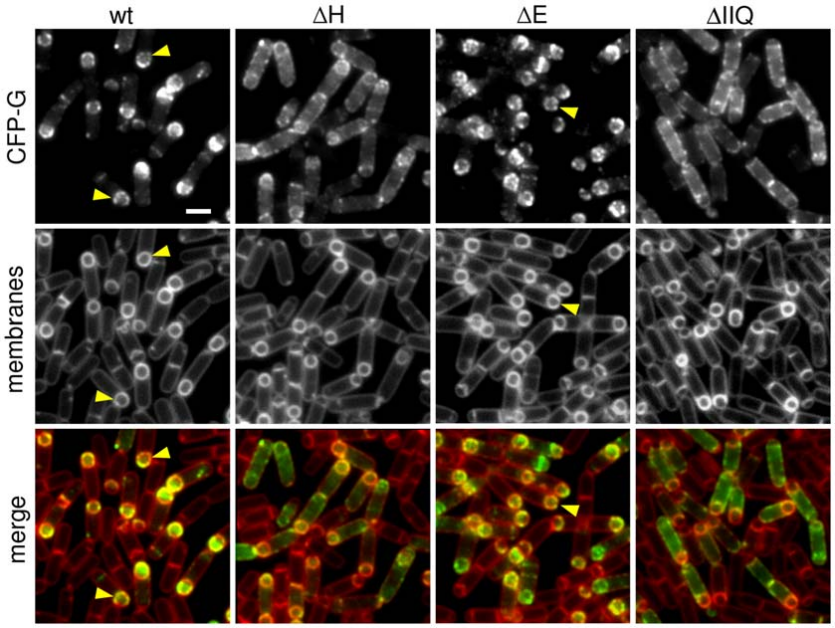
CFP-SpoIIIAG localization to the forespore membranes requires SpoIIIAH and SpoIIQ. CFP-SpoIIIAG localization (false-colored green in the lower panel) was visualized by fluorescence microscopy at hour 2 of sporulation in a wild-type background (wt, BTD49), in a Δ*spoIIIAH* mutant (ΔH, BCM575), a Δ*spoIIIAE* mutant background (ΔE, BCM810), and a Δ*spoIIQ* mutant (ΔIIQ, BCM736). The membranes from the same field were visualized with the fluorescent dye TMA-DPH (false-colored red in the lower panel). Carets highlight the punctate CFP-SpoIIIAG localization pattern. Scale bar, 1 µm.

Previous studies of SpoIIIAH and SpoIIQ indicate that these proteins localize to the membranes that surround the forespore [16],[20]. Our co-immunoprecipitation data suggest that the other SpoIIIA proteins reside in a complex with SpoIIIAH and SpoIIQ, and should therefore also be present in these membranes. To investigate this, we analyzed the sub-cellular localization of SpoIIIAG using a CFP-SpoIIIAG fusion. The gene fusion was placed under the control of the native *spoIIIA* promoter and inserted at a non-essential locus in the genome. The fusion partially complemented a *spoIIIAG* mutant (2% sporulation efficiency, 1000-fold better than a *spoIIIAG* null strain). Analysis of CFP-SpoIIIAG by fluorescence microscopy at hour 2 of sporulation revealed that the protein specifically localizes to the engulfing septal membranes (Figure 3). Moreover, the CFP-SpoIIIAG fusion had a punctate staining pattern similar to what has been observed previously for SpoIIIAH and SpoIIQ [16],[19].

To determine whether this localization pattern requires SpoIIQ, SpoIIIAH or the other SpoIIIA proteins, we analyzed CFP-SpoIIIAG in strains lacking these proteins. In the absence of SpoIIIAH, SpoIIIAG was mislocalized (Figure 3). CFP-SpoIIIAG was present in all the cytoplasmic membranes of the mother cell; however, there was still some enrichment of the fusion protein in the membranes surrounding the forespore. Interestingly, proper localization of SpoIIIAG did not require the other SpoIIIA proteins. The absence of SpoIIIAA or SpoIIIAE had no impact on CFP-SpoIIIAG localization (Figure 3 and data not shown). Moreover, the mislocalization of CFP-SpoIIIAG in cells lacking the entire *spoIIIA* operon was indistinguishable from the SpoIIIAH mutant (data not shown). Finally, in the absence of the forespore membrane protein SpoIIQ, CFP-SpoIIIAG had the most severe mislocalization phenotype with virtually no enrichment of the protein in the forespore membranes (Figure 3). Altogether, these data provide further support for the idea that SpoIIIAG and the rest of the SpoIIIA proteins reside in a multimeric membrane complex with SpoIIIAH and SpoIIQ. Moreover, they suggest that SpoIIIAH serves as an important link between SpoIIQ and the rest of the SpoIIIA complex.

### The SpoIIIA and SpoIIQ proteins are required for integrity of the forespore

**Figure 4:**
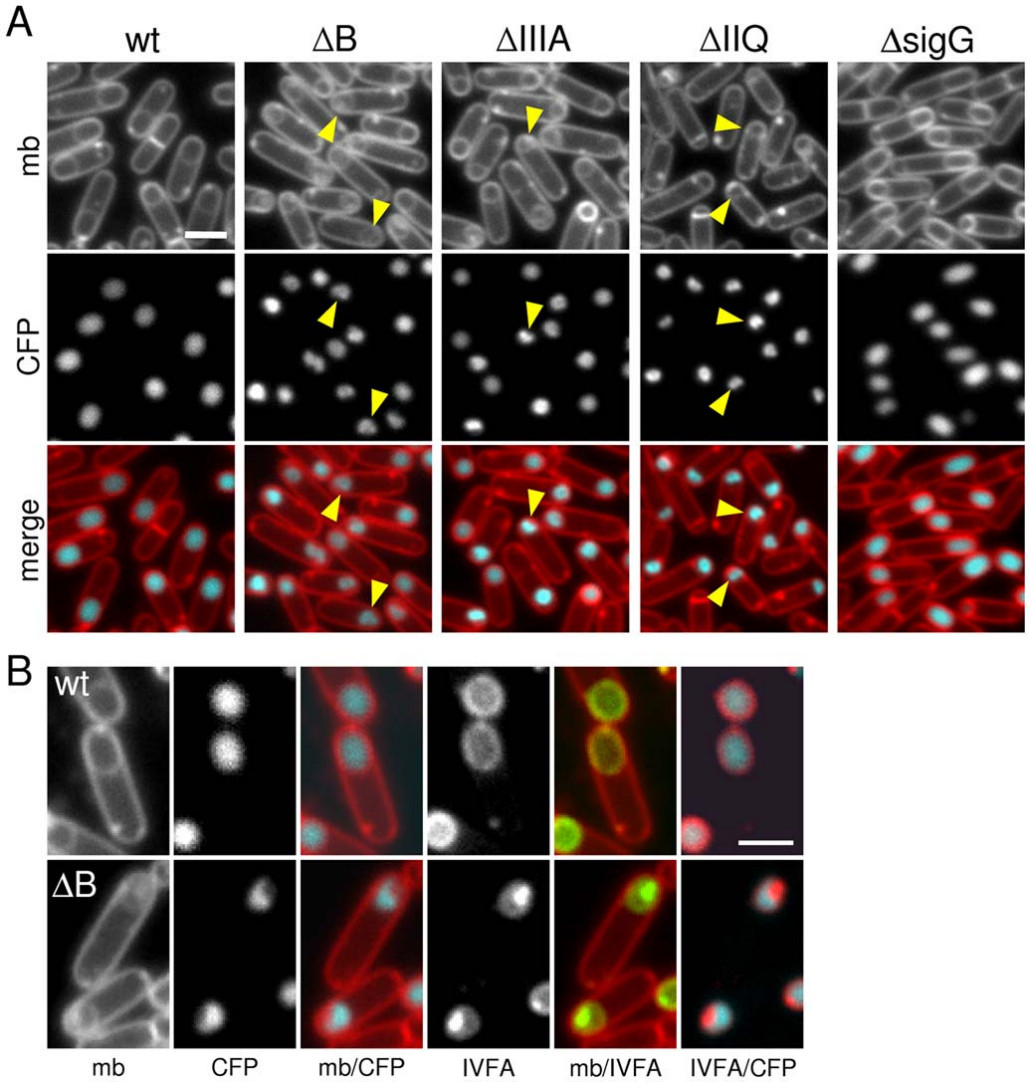
Morphological defects in the absence of SpoIIIA or SpoIIQ. (A) Forespore morphology was monitored by fluorescence microscopy at hour 3 of sporulation in a wild-type background (wt, BCM703), a Δ*spoIIIAB* mutant (ΔB, BCM704), a Δ*spoIIIA* mutant (ΔIIIA, BCM706), a Δ*spoIIQ* mutant (ΔIIQ, BCM716), and in a strain lacking σ^G^ (ΔsigG, BCM708). All strains contained a forespore reporter (P*_spoIIQ_-cfp*; false-colored blue in the lower panel) to visualize the forespore cytoplasm. The membranes (mb) from the same field were visualized with the fluorescent dye TMA-DPH (false-colored red in the lower panel). Carets highlight examples of “collapsed” forespores. (B) Larger images highlighting the cytoplasmic CFP signal and the localization YFP-SpoIVFA (IVFA; false-colored green) in wild-type (wt, BCM703) and a Δ*spoIIIAB* mutant (ΔB, BCM704). Larger images of all strains can be found in Figure S5. Scale bar, 1 µm.

In the course of our analysis of the SpoIIIA mutants, we discovered that the SpoIIIA proteins play an important role in maintaining forespore integrity after engulfment is complete. During the process of engulfment, cells lacking the SpoIIIA proteins appear similar to wild-type (Figure 3) [31]. However, upon completion of engulfment, the SpoIIIA mutants exhibited morphological defects: the forespores appeared smaller and in many cases collapsed (Figure 4). To more carefully analyze this new phenotype, we used a forespore-specific CFP reporter to label the forespore cytoplasm and a YFP-SpoIVFA fusion that localizes to the mother-cell membranes that surround the forespore [20],[32]. In wild-type cells at hour 3 of sporulation when engulfment was complete, the CFP reporter appeared homogenous in the forespore cytoplasm and the YFP-SpoIVFA fusion labeled the forespore membranes in a smooth and continuous fashion (Figure 4 and Figure S5). By contrast, in the SpoIIIA mutants the cytoplasmic CFP signal in the forespore appeared patchy and distorted (Figure 4A). Moreover, the YFP-SpoIVFA fusion localized in bright patches around the forespore (Figure 4B and Figure S5). The merged images revealed that the patchy YFP-SpoIVFA signal was coincident with regions of the forespore that lacked cytoplasmic CFP (Figure 4B and Figure S5). These results suggest that in the absence of the SpoIIIA proteins, the engulfed forespore membranes collapse, forming large invaginations. A similar collapsed forespore phenotype was observed in the absence of SpoIIQ and in a Walker B mutant of the SpoIIIAA protein (Figure 4A and data not shown). Importantly, the forespores in cells lacking σ^G^ did not display this phenotype and were indistinguishable from wild-type (Figure 4 and Figure S5). Thus, the failure to maintain proper forespore morphology is not due to the inability to activate σ^G^. Instead, these results are consistent with a model in which the SpoIIIA-SpoIIQ complex as well as the ATPase activity of SpoIIIAA are necessary to maintain proper forespore integrity.

**Figure 5:**
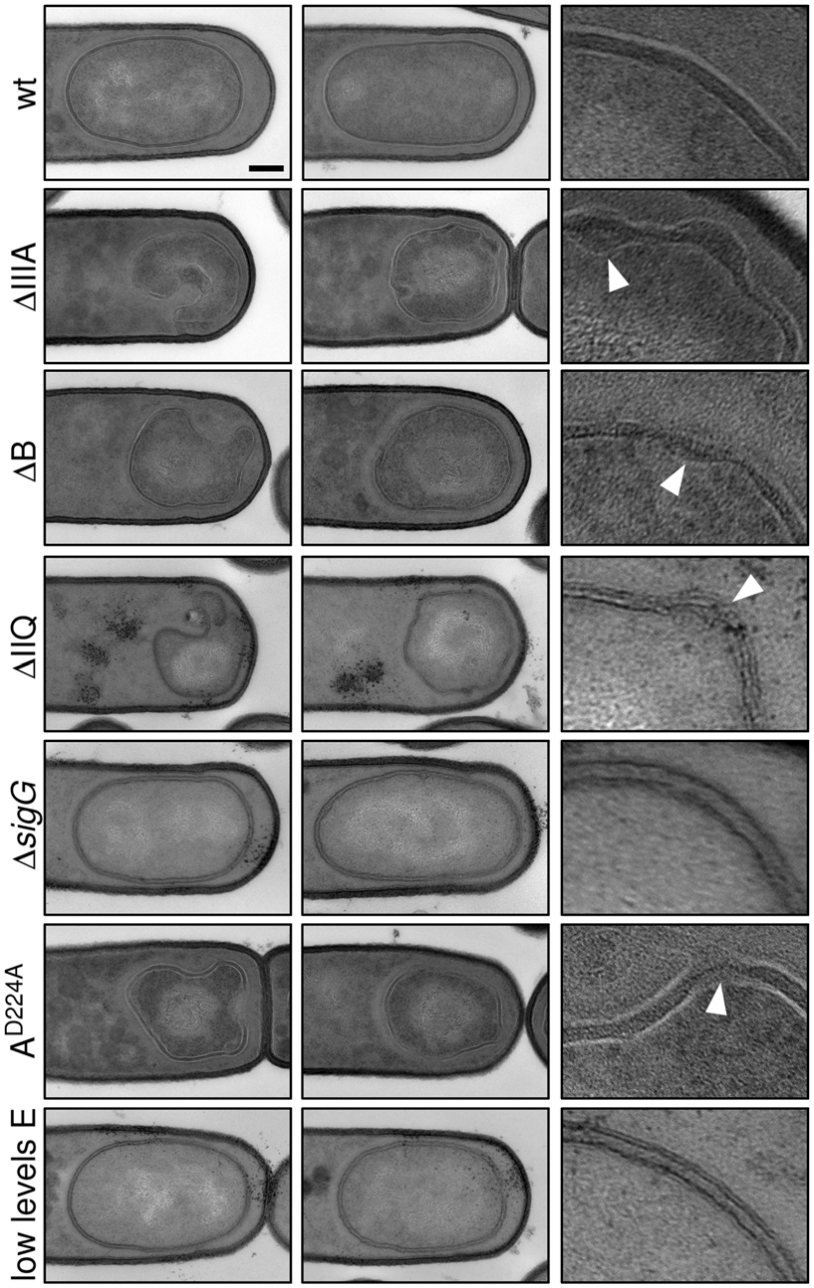
Morphological defects in the absence of the SpoIIIA or SpoIIQ proteins. Forespore morphology was assessed by electron microscopy at hour 3 of sporulation in wild-type (wt, PY79), a Δs*poIIIA* mutant (ΔIIIA, BDR841), a Δ*spoIIIAB* mutant (ΔB, BTD119), a Δs*poIIQ* mutant (ΔIIQ, BTD141), a Δ*sigG* mutant (*Δ*sigG, BDR104), a *spoIIIAA* ^D224A^ ATPase mutant (A^D224A^, BTD2683), and a strain that contains low levels of SpoIIIAE (low E, BTD3019). For each strain, two typical forespores are shown in the first two columns. Scale bar, 200 nm. The last column shows a 5× enlargement of the forespore membranes. The carets highlight bulged or ruptured membranes in the mutants.

To more closely compare the collapsed forespores in the various mutants, we analyzed their morphology by electron microscopy. Cells at hour 3 of sporulation were fixed, embedded, sectioned, and stained using standard protocols (see Material and Methods). Analysis of sporulating cells by electron microscopy revealed several morphological defects in the mutants. Consistent with our observations using fluorescence microscopy, the forespores were significantly smaller than wild-type and many displayed large invaginations (Figure 5). Analysis of forespores in strains lacking SpoIIIAA, SpoIIIAB, SpoIIIAE or SpoIIQ revealed that 30-52% (n = 50 per mutant) displayed membrane invaginations. Finally, the two membranes that surround the forespore had constrictions and bulges and in some cases appeared to have ruptured (carets in Figure 5). By contrast, the size and morphology of the forespores in the σ^G^ mutant were indistinguishable from wild-type as observed previously [6]. Importantly, the phenotypes of the SpoIIIA mutant forespores (including the SpoIIIAA ATPase mutant) and the SpoIIQ mutant forespores were comparable (Figure 5), suggesting a defect in a shared function.

All together, these results suggest that the SpoIIIA-SpoIIQ complex (and the ATPase activity of SpoIIIAA) is required to maintain the integrity of the forespore. Importantly, since forespore integrity did not require σ^G^, these results indicate that forespore collapse in the SpoIIIA and SpoIIQ mutants is not a consequence of the failure to activate σ^G^ (and the late program of forespore gene expression under its control). Instead, these results suggest that the block to σ^G^ activity is a manifestation of the failure to maintain forespore integrity.

### Low levels of SpoIIIA proteins can support efficient sporulation

How might the SpoIIIA-SpoIIQ complex maintain forespore integrity? The localization patterns of SpoIIQ and the SpoIIIA proteins [16],[19] suggest that the SpoIIIA-SpoIIQ complex could function in a structural capacity. These complexes are distributed throughout the forespore membranes in what appears to be helical arcs [16],[19] and could play a cytoskeletal role in maintaining or reinforcing the architecture of the spore. On the other hand, the similarity of the SpoIIIA proteins to components of specialized secretion systems and the requirement of the ATPase domain of SpoIIIAA for proper forespore morphology argue in favor of an enzymatic function for the complex. For example, if the SpoIIIA-SpoIIQ complex functions as a secretion apparatus, its role in maintaining forespore integrity could be to nurture the forespore by secreting nutrients or osmolytes such as ions, metabolites or small proteins into the forespore as has been proposed by Camp and Losick [21],[23].

**Figure 6:**
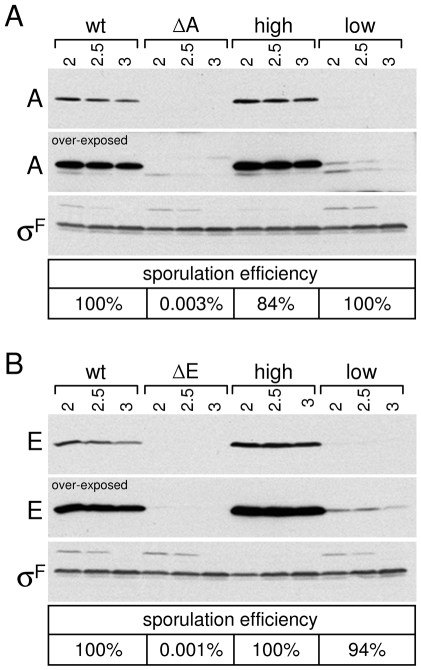
Low levels of SpoIIIAA or SpoIIIAE support efficient sporulation. Immunoblot analysis of whole cell lysates from sporulating cells. (A) Comparison of SpoIIIAA (A) levels in wild-type (wt, PY79), a Δ*spoIIIAA* mutant (ΔA, BTD117), a strain expressing high levels of SpoIIIAA (high, BTD2341), and a strain expressing low levels of SpoIIIAA (low, BTD3023). (B) Comparison of SpoIIIAE levels (E) in wild-type (wt, PY79), a Δ*spoIIIAE* mutant (ΔE, BTD113), a strain expressing high levels of SpoIIIAE (high, BTD2349), and a strain expressing low levels of SpoIIIAE (low, BTD3019). The levels of σ^F^ are similar in all lysates indicating that all strains entered sporulation with similar efficiency. Short and long (over-exposed) exposures of the anti-SpoIIIAA (A) and anti-SpoIIIAE (E) immunoblots are shown. Time (in hours) after the initiation of sporulation is shown. Sporulation efficiencies of the same strains are shown below the immunoblots.

We reasoned that if the SpoIIIA-SpoIIQ complex plays a structural role in forespore integrity, then reduction in the levels of proteins in the complex might result in a collapsed forespore phenotype and impaired sporulation. By contrast, if this complex plays an enzymatic function during spore maturation, a reduction in protein levels might not significantly affect spore morphogenesis. To investigate this, we generated strains that produced low levels of SpoIIIAA, or SpoIIIAE (see Material and Methods). As shown in Figure 6, the levels of SpoIIIAA and SpoIIIAE in these strains were barely detectable by immunoblot. Serial dilution of the extract from wild-type cells suggests that the levels of SpoIIIAA and SpoIIIAE are 5-10-fold reduced (data not shown). Importantly, despite these low levels, the sporulation efficiency of these strains was similar to wild-type (Figure 6). Moreover, examination of the forespores by fluorescence and electron microscopy indicates that low levels of SpoIIIAA and SpoIIIAE do not affect forespore integrity (Figure 5 and Figure S6). Similar results were obtained using a strain that produced low levels of SpoIIIAF (data not shown). These results suggest that the SpoIIIA-SpoIIQ complex has an enzymatic rather than a structural role in maintaining proper forespore integrity.

### SpoIIIA and SpoIIQ are not required for early σ^G^ activity but are needed to maintain late σ^G^ activity

Collectively, our results are consistent with the feeding-tube model proposed by Camp and Losick [21],[23] in which the putative SpoIIIA-SpoIIQ secretion apparatus secretes nutrients and/or osmolytes into the forespore, “feeding” the spore in its final stages of preparation for dormancy. In the context of this model, the role of the SpoIIIA proteins and SpoIIQ in σ^G^ activation is not to secrete a specific signal into the forespore to trigger transcription factor activation. Instead, the “feeding” of the forespore is itself what allows σ^G^-dependent gene expression. In this scenario, the lack of σ^G^ activity in the SpoIIQ and SpoIIIA mutants is a consequence of the loss of metabolic potential in the forespore. This model is extremely compelling in its parsimony but is a significant departure from current thinking.

All current models for σ^G^ regulation involve an unidentified inhibitor that holds the sigma factor inactive during the engulfment process. Relief of inhibition is then triggered upon the completion of engulfment and/or by a signal that is received from the mother cell. The data in support of such an inhibitor comes from experiments in which the *sigG* gene was fused to a strong σ^F^-responsive promoter (P*_spoIIQ_*) that is active immediately after polar division. (The *sigG* gene is itself recognized by σ^F^ but σ^F^-dependent transcription of *sigG* occurs only after a 30-minute delay [8],[10].) Cells harboring the P*_spoIIQ_*-*sigG* fusion synthesize σ^G^ prematurely, prior to the completion of engulfment. However, despite premature synthesis, the timing of σ^G^ activity is indistinguishable from wild-type and is detectable only upon the completion of engulfment [2],[9],[21],[33],[34]. Thus, these data suggest that σ^G^ is held inactive until a signal from the mother cell and/or the completion of engulfment triggers relief of inhibition.

**Figure 7:**
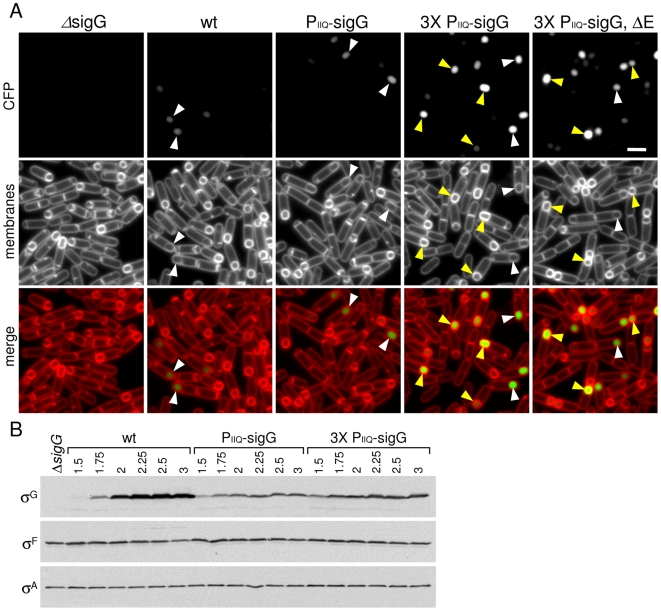
σ^G^ is active when synthesized prior to the completion of engulfment. (A) σ^G^ activity was assessed by microscopy using a fluorescence reporter (P*_sspE_-gfp*; false-colored green in the lower panel) in a Δ*sigG* mutant (ΔsigG, BTD3004), a wild-type background (wt, BTD3002), a Δ*sigG* mutant containing one copy of P*_spoIIQ_-sigG* (P*_IIQ_-sigG*, BTD3007), or three copies of P*_spoIIQ_-sigG* (3× P*_IIQ_-sigG*, BCM791), and a Δ*sigG*, Δ*spoIIIAE* double mutant that contains three copies of P*_spoIIQ_-sigG* (ΔE, BCM816). Sporulating cells were monitored at hour 2 of sporulation. The membranes from the same field were visualized using the dye TMA-DPH (false-colored red) and merged with the GFP signal. The membrane dye inefficiently traverses the lipid bilayer and therefore reports on the engulfment status of the forespore [54]. Forespores that stain weakly with TMA-DPH (white carets) have been completely engulfed by the mother cell. Forespores that have not yet completed engulfment have strong signal due to the two membranes surrounding the spore. Forespore that have σ^G^ activity but have not completed engulfment are indicated (yellow carets). Scale bar, 1 µm. Similar results were obtained with a P*_sspB_*-*gfp* reporter (not shown). (B) Immunoblot analysis of whole cell lysates from sporulating cells. The levels of σ^G^ were analyzed in sporulating cells from the same strains described in A. σ^F^ levels were monitored to control for efficiency of sporulation. σ^A^ levels were monitored to control for loading.

The compelling nature of the feeding-tube model prompted us to re-visit this important experiment that challenges its simplicity. Examination of σ^G^ protein levels in a sporulation time course using one of the original P*_spoIIQ_*-*sigG* fusions [34] revealed premature synthesis of σ^G^ as reported previously (Figure 7B). However, the levels of σ^G^ protein present prior to the completion of engulfment (hour 1.5–2) were quite low. In fact, at hour 2, the level of σ^G^ in wild-type was slightly *higher* than the strain carrying the P*_spoIIQ_-sigG* fusion (Figure 7B). Although the P*_spoIIQ_*-directed accumulation of σ^G^ may vary with the exact construct used or the medium employed to induce sporulation [33],[34], these results suggest that σ^G^ levels in the P*_spoIIQ_-sigG* fusion strain might have been too low for the transcriptional reporter to reveal early activity. Thus, these data raised the possibility that σ^G^ could be active prior to the completion of engulfment and not subject to inhibition.

To investigate this idea, we increased the levels of premature σ^G^ by introducing three copies of the P*_spoIIQ_*-*sigG* fusion at three non-essential loci in the genome (see Material and Methods). Under these conditions, the levels of σ^G^ were approximately 2-fold higher at early time points (hour 1.5–1.75) of sporulation (Figure 7B). Strikingly, in this strain, we could easily detect premature σ^G^ activity. Specifically, a σ^G^-responsive promoter (P*_sspE_*) fused to *gfp* revealed fluorescence in the majority of cells that had not yet completed engulfment (Figure 7A). Importantly, early σ^G^ activity was independent of SpoIIQ, SpoIIIA and mutants blocked in engulfment (Figure 7A and , see yellow carets). Moreover, consistent with the idea that the SpoIIIA-SpoIIQ complex is required to maintain metabolic potential in the forespore, at late time points (T3.5), the intensity of GFP fluorescence in the SpoIIIA and SpoIIQ mutants was lower than in the matched wild-type control (Figure S7B). Thus, in the absence of SpoIIIA or SpoIIQ, we could detect early σ^G^ activity but at late stages of sporulation, σ^G^-dependent gene expression was reduced, suggesting that it may have ceased. These results challenge the idea that there is an active mechanism holding σ^G^ inactive that is relieved upon the completion of engulfment (and/or by a signal received from the mother cell). Furthermore, they provide support for the idea that the role of SpoIIIA-SpoIIQ complex in σ^G^ activity is to maintain metabolic potential in the forespore.

## Discussion

**Figure 8:**
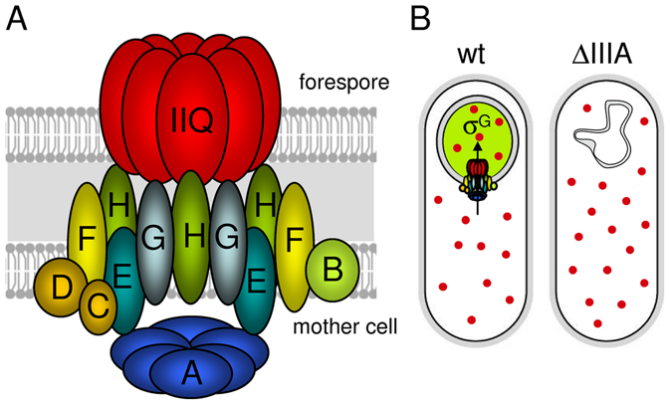
The SpoIIIA proteins in the mother cell and SpoIIQ in the forespore form a secretion complex. (A) Schematic diagram showing the SpoIIIA-SpoIIQ secretion complex in the two membranes that surround the forespore. SpoIIIAA (A), SpoIIIAB (B), SpoIIIAC (C), SpoIIIAD (D), SpoIIIAE (E), SpoIIIAF (F), SpoIIIAG (G), SpoIIIAH (H), SpoIIQ (Q) are shown. SpoIIIAA (A) is shown as a hexamer by analogy to other traffic ATPases [25]. SpoIIQ (Q) is shown as a multimeric pore based on the experiments of Meisner et al [22]. The actual stoichiometry of proteins in the complex is unknown. (B) The feeding-tube model. In wild-type cells (wt), the SpoIIIA-SpoIIQ complex secretes an unknown metabolite/osmolyte (red circle) into the forespore that maintains forespore integrity and σ^G^ activity (indicated by a green forespore). In the absence of the SpoIIIA (ΔIIIA) or SpoIIQ proteins, the forespore looses metabolic potential; the forespore collapses and σ^G^ activity cannot be maintained.

We have presented evidence that the SpoIIIA proteins in the mother cell and SpoIIQ in the forespore form a large multimeric complex that spans the two membranes that surround the forespore (Figure 8A). In addition, we have shown that the ATPase motifs in SpoIIIAA are required for SpoIIIAA function *in vivo*. Based on these findings and the recent work of Camp and Losick [21],[22] and Moran and colleagues [35], we propose that the SpoIIIA-SpoIIQ complex forms a novel secretion apparatus that links the mother cell and forespore. This putative secretion complex appears to have been cobbled together from components that are found in a variety of specialized secretion systems found in Gram-negative bacteria. SpoIIIAA resembles ATPases found in type II and type IV secretion systems. SpoIIIAB shares weak similarity with the GspF and TadB/C proteins found in type II and type IV secretion systems, respectively [36],[37]. SpoIIIAH shares homology with the YscJ/FliF protein family found in type III secretion systems [21],[22]. SpoIIIAG also shares weak similarity (albeit weaker than SpoIIIAH) to the YscJ/FliF protein family [22]. SpoIIIAF shares weak similarity with FlhB (YscU) found in type III secretion systems [22]. Finally, SpoIIIAE is similar to ABC-type permeases involved in Type I secretion.

Further support for the idea that these proteins form a secretion apparatus comes from recent findings that the extracellular domains of SpoIIIAH and SpoIIQ, that reside in the space between the mother cell and forespore membranes, are accessible to modification by a soluble biotin ligase produced in the forespore [22]. These results suggest that SpoIIQ forms a pore in the forespore membrane that allows the ligase access to the extracellular domains of SpoIIIAH and SpoIIQ. One unresolved issue concerning this putative secretion complex is the role of SpoIIIAH. SpoIIIAH appears to lie at the heart of the complex linking the SpoIIIA proteins in the mother cell to SpoIIQ in the forespore. Paradoxically, cells lacking SpoIIIAH have a sporulation efficiency of 5% while all other mutants in the complex are 1,000-fold worse (Table S1). Apparently, in the absence of SpoIIIAH, the other SpoIIIA proteins can assemble a partially functional complex with SpoIIQ. One possibility is that SpoIIIAH is principally involved in tethering the other SpoIIIA proteins to SpoIIQ and that in its absence they can still interact, albeit weakly, with the forespore membrane protein. Alternatively, the SpoIIIAG protein, which, like SpoIIIAH, shares weak homology with the YscJ/FliF protein family [35], might function in place of SpoIIIAH. Either model is consistent with our findings that CFP-SpoIIIAG remains somewhat enriched in the membranes that surround the forespore in the absence of SpoIIIAH and that this enrichment is lost in the SpoIIQ mutant (Figure 3).

Our data also indicate that the SpoIIIA proteins and SpoIIQ are required to maintain forespore integrity. In the absence of these proteins, the forespore appears normal at early stages of sporulation. However, at a time when engulfment is complete, the forespore develops large invaginations and appears to shrink and/or collapse. The collapse of the forespore membranes likely explains the instability of the forespore [38],[39] and the loss of compartmentalized gene expression previously observed at late times in development in SpoIIIA mutants [33],[40]. We hypothesize that these phenotypes are due to lack of osmolytes and/or loss of metabolic potential. Indeed, it has been shown previously that mutations that impair glycerol metabolism in *B. subtilis* result in membrane collapse [41] and that carbon starvation leads to cell autolysis [42]. Moreover, defects in osmotolerance in *Listeria monocytogenes* affects cell morphology [43]. Finally, our data suggest that the role of SpoIIIA in maintaining forespore integrity is enzymatic rather than structural. Collectively, these results support and extend the feeding-tube model proposed by Camp and Losick [21],[23], in which the SpoIIIA-SpoIIQ secretion apparatus secretes nutrients and/or osmolytes into the forespore, “feeding” the spore in the final stages of preparation for dormancy (Figure 8B).

In this model, the maintenance of metabolic potential in the forespore is what enables σ^G^ activity. Our data showing that there is not an active mechanism holding σ^G^ inactive that is relieved upon the completion of engulfment provides further support for this new model. It is noteworthy that Stragier and colleagues have recently proposed that σ^G^ activity is held inactive until engulfment is complete by a protein (Gin/CsfB) synthesized in the forespore under the control of σ^F^ [34]. Although this model has been challenged [21],[44], our data do not rule out the possibility that Gin could serve as a timing device to help prevent early σ^G^ activity as was originally proposed by Piggot [44]. For example, Gin could be a saturable inhibitor of σ^G^. Once σ^G^ levels (synthesized under the control of σ^F^) exceed this negative regulator and provided the forespore has metabolic potential, σ^G^ could flip its auto-regulatory switch. In support of this idea, the level of premature σ^G^ activity was ∼2-fold higher in the strain harboring three copies of P*_spoIIQ_*-*sigG* when Gin was absent (Figure S8).

One outstanding question in the regulation of σ^G^ is how its activity is coupled to proper engulfment. Our data showing that premature synthesis of σ^G^ results in early σ^G^ activity even when engulfment is impaired suggests that a separate regulatory pathway that links σ^G^ activity and engulfment is unlikely. Instead, we favor the model that SpoIIIA and SpoIIQ proteins fail to assemble into an active complex when engulfment is perturbed and this prevents σ^G^ activity. In this model, the assembly of an active complex serves as the “surveillance mechanism” that couples gene expression to proper engulfment [17]. Interestingly, we find that in engulfment mutants, a subset (11%) of the sporulating cells succeed in activating σ^G^ to levels comparable to wild-type (Figure S9). We interpret this class of cells as those that have successfully assembled an active SpoIIIA-SpoIIQ secretion complex and therefore can maintain forespore physiology and σ^G^ activity despite the failure to engulf. Consistent with this idea, the subset of cells with σ^G^ activity was completely eliminated in a SpoIIIAA^D224A^ ATPase mutant or in the absence of SpoIIQ or the SpoIIIA proteins (Figure S9 and data not shown).

A secretion apparatus that allows the mother cell to feed the forespore could have arisen because the forespore becomes fully engulfed inside the mother cell and therefore isolated from the external environment. In addition, the forespore developmental program might involve the down-regulation (or shut-off) of genes required for certain metabolic functions [45]. Under both conditions, the forespore would become dependent on the mother cell for nutrients and/or osmolytes. The observation that in engulfment mutants the forespore remains in contact with the environment yet most forespores lack σ^G^ activity [8],[13],[14] (Figure S9) suggests that the forespore has lost the ability to provide for itself and has become dependent on the mother cell for its final stages of maturation.

In conclusion, our results and those of Camp and Losick are most consistent with a model in which the SpoIIIA-SpoIIQ complex functions as a novel secretion complex allowing the mother cell to nurture the forespore at late stages in sporulation (Figure 8B). In this model, σ^G^ activity depends on this complex not for an activating signal but to maintain forespore physiology. Our data suggest that the principal regulation of σ^G^ activity is i) the delay in *sigG* transcription under the control of σ^F^; ii) the saturable inhibitor Gin that prevents pre-mature σ^G^ activity; iii) the auto-activation of the *sigG* gene; and iv) the maintenance of metabolic potential by the SpoIIIA-SpoIIQ complex. Moreover, our results provide a plausible explanation for the coupling of σ^G^ activity to proper engulfment. Finally, the feeding-tube model nicely accounts for why no signaling protein required for σ^G^ activation has ever been identified and why no mutant has been found that can bypass the requirement for SpoIIQ or the SpoIIIA proteins in compartmentalized σ^G^ activity. The challenge for the future is to identify the factor or factors secreted by the SpoIIIA-SpoIIQ complex that nurture the forespore in its final stages of development.

## Materials and Methods

### General methods

All *B. subtilis* strains were derived from the prototrophic strain PY79 [46]. Sporulation was induced by resuspension at 37°C according to the method of Sterlini-Mandelstam [47] or by exhaustion (in supplemented DS medium; [48]). Sporulation efficiency was determined in 36-h cultures as the total number of heat-resistant (80°C for 20 min) colony forming units (CFUs) compared with wild-type heat-resistant CFUs. *B. subtilis* strains harboring the P*_spoIIQ_*-*sigG* fusions were constructed by direct transformation of ligation products because *sigG* is toxic to *E. coli*. Whole cell lysates from sporulating cells (induced by resuspension) were prepared as described previously [49]. Samples were heated for 10 min at 50°C prior to loading. Equivalent loading was based on OD_600_ at the time of harvest. Tables and of strains, plasmids and oligonucleotide primers and descriptions of plasmid construction can be found online as supplementary data (Table S2, Table S3, Table S4; Text S1).

### Protein purification and antibody production

His_6_-SpoIIIAA, His_6_-SpoIIIAF and His_6_-SpoIIIAG fusion proteins were expressed in *E. coli* BL21 DE3 pLysS and purified on Ni^2+^-NTA agarose (Qiagen). GST-SpoIIIAE fusion protein was expressed in *E. coli* NB42 and purified on glutathione-agarose (GE Life Sciences). A complete description of the purifications can be found in Text S1. Peak fractions were pooled and used to generate rabbit polyclonal antibodies (Covance). Crude serum was affinity-purified as described [50].

### Antibody resins preparation

All antibody resins were prepared as described previously [30]. Briefly, affinity-purified antibodies (2 mg) were batched absorbed to 1 ml protein A-sepharose (Amersham). The antibody resin was washed with phosphate buffered saline (PBS) and covalently cross-linked to the protein A-sepharose by the addition of Disuccinimidyl Suberate (Pierce) to a final concentration of 5 mM. After 30 minutes the reaction was quenched by the addition of Tris pH 7.5 to a final concentration of 100 mM. The antibody resin was washed with 100 mM Glycine pH 2.5 to remove uncrosslinked antibody and then neutralized with 1× PBS.

### Co-immunoprecipitation from detergent solubilized membrane fractions

Preparation of crude membranes and detergent solubilization of membrane proteins was performed as described [30]. 50 ml cultures were harvested at hour 2.5 of sporulation (by resuspension) and washed two times with 1× SMM (0.5 M Sucrose, 20 mM MgCl_2_, 20 mM Maleic acid pH 6.5) at room temperature. Cells were resuspended in 1/10 volume 1× SMM and treated with Lysozyme (0.5 mg/ml). Protoplasts were collected by centrifugation and flash frozen in N_2_(l). Thawed protoplasts were disrupted by osmotic lysis with 3 ml hypotonic buffer (Buffer H) (20 mM Hepes pH 8, 200 mM NaCl, 1 mM DTT, with protease inhibitors: 1 mM PMSF, 0.5 µg/ml leupeptin, 0.7 µg/ml pepstatin). MgCl_2_ and CaCl_2_ were added to 1 mM and lysates were treated with DNAseI (10 µg/ml) (Worthington) and RNAseA (20 µg/ml) (USB) for 30 min on ice. The membrane fraction was separated by centrifugation at 100,000×g for 1 hour at 4°C. The supernatant was carefully removed and the membrane pellet was dispersed in 200 µl Buffer G (Buffer H with 10% Glycerol). Crude membranes were aliquoted and flash frozen in N_2_(l).

Crude membranes were diluted 5-fold with Buffer S [Buffer H with 20% Glycerol and 100 µg/ml Lysozyme] and membrane proteins were solubilized by the addition of the nonionic detergent DDM (n-dodecyl-β-d-maltopyranoside, Sigma) to a final concentration of 0.5%. The mixture was rotated at 4°C for 1 hour. Soluble and insoluble fractions were separated by centrifugation at 100,000×g for 1 hour at 4°C. The soluble fraction (the load) was mixed with 40 µl antibody resin and rotated for 4 h at 4°C. The resin was pelleted at 5 Krpm and the supernatant (the flow through) was removed. The resin was washed 4 times with 1 ml Buffer S containing 0.01% DDM. Immunoprecipitated proteins were eluted by the addition of 90 µl of Sodium Dodecyl Sulfate (SDS) sample buffer and heated for 15 minutes at 50°C. The eluted material (the IP) was transferred to a fresh tube and 2-Mercaptoethanol was added to a final concentration of 10%. The load, flow through and IP were analyzed by immunoblot.

### Mass spectrometry analysis

Immunoprecipitates were trypsinized and the peptides were separated on a nanoscale C18 reverse-phase high-pressure liquid chromatography capillary column, and were subjected to electrospray ionization followed by MS using an LCQ DECA ion-trap mass spectrometer.

### Immunoblot analysis

Proteins were separated by SDS-PAGE on 15% polyacrylamide gels, electroblotted onto Immobilon-P membranes (Millipore) and blocked in 5% nonfat milk in phosphate-buffered saline (PBS)-0.5% Tween-20. The blocked membranes were probed with affinity-purified anti-SpoIIIAA (1∶10,000), anti-SpoIIIAE (1∶5,000), anti-SpoIIIAF (1∶10,000), anti-SpoIIIAG (1∶10,000), anti-spoIIIAH (1∶10,000) [30], anti-SpoIID (1∶10,000) [49], anti-SpoIIQ (1∶10,000) [49], anti-GFP (1∶10,000) [32], anti-myc (1∶1000) (Covance), anti-σ^G^ (1∶20,000) (a gift from M. Ho and R. Losick), anti-σ^F^ (1∶5,000) [51], and anti-σ^A^ (1∶10,000) [52]. The primary antibodies were diluted into 3% BSA in 1× PBS-0.05% Tween-20. Primary antibodies were detected using horseradish peroxidase-conjugated goat, anti-rabbit G (BioRad) and the Western Lightning reagent kit as described by the manufacturer (PerkinElmer).

### Fluorescence microscopy

Fluorescence microscopy was performed with an Olympus BX61 microscope as previously described [32]. Fluorescent signals were visualized with a phase contrast objective UplanF1 100× and captured with a monochrome CoolSnapHQ digital camera (Photometrics) using Metamorph software version 6.1 (Universal Imaging). Exposure times were typically 500 ms for CFP and GFP. The membrane dye TMA-DPH (Molecular Probes) was used at a final concentration of 0.01 mM and exposure times were typically 200 ms. Images were analysed, adjusted and cropped using Metamorph software. The cells were concentrated by centrifugation (8 K rpm for 30 sec) prior to visualization. This step had no impact on the reported phenotypes.

### Electron microscopy

Samples were collected at hour 3 of sporulation (by resuspsension) and prepared for electron microscopy. Cells were resuspended in PBS and fixed (0.5% paraformaldehyde and 2.5% glutaraldehyde) for 4 h at room temperature followed by dehydration with acetone and embedding in Epon resin. Finally, ultrathin sections were stained with lead citrate. For evaluation of forespore morphology, only longitudinal sections were considered. Greater than 50 sporangia were examined per strain.

## Supporting Information

Figure S1 Sequence alignment of the C-terminal domain of SpoIIIAA (IIIAA) from *B. subtilis* with the ATPase domains of several secretion ATPases. The conserved motifs (red boxes) found in all secretion NTPases are highlighted. Mutated residues are indicated (blue asterisk). The alignment was made using ClustalW (http://www.ch.embnet.org/software/ClustalW.html) and BOXSHADE (http://www.ch.embnet.org/software/BOX_form.html).
(0.96 MB TIF)

Figure S2 The ATPase motifs in SpoIIIAA are required for σ^G^ activity and sporulation efficiency. (A) σ^G^ activity was assessed in single cells by microscopy using a fluorescent reporter (P*sspE-cfp*) in a wild-type background (wt, BTD1609), a δ*spoIIIAA* mutant (δA, BTD2713), a δ*spoIIIAA* mutant containing a wild-type copy of *spoIIIAA* inserted at a non-essential locus (A(wt), BTD2719), a *spoIIIAA* ^D224A^ Walker B box point mutant (A^D224A^, BTD2775), a *spoIIIAA* ^K149A^ Walker A box point mutant (A^K149A^, BTD2906), a *spoIIIAA* ^E180Q^ Asp box point mutant (A^E180Q^, BTD2907), and a *spoIIIAA* ^H250Y^ His box point mutant (A^H250Y^, BTD2909). Cells were visualized at hour 3 of sporulation. Forespore CFP fluorescence (false-colored green in the lower panel) and the fluorescent membrane dye TMA-DPH (false-colored red) are shown. Scale bar, 1 µm. Sporulation efficiencies of the same strains are shown below the fluorescent images. (B) Immunoblot analysis of whole cell lysates from sporulating cells shown in A. Time (in hours) after the initiation of sporulation is indicated.
(0.78 MB TIF)

Figure S3 The SpoIIIAE, SpoIIIAF and SpoIIIAG proteins reside in a membrane complex. Immunoprecipitations were performed on detergent-solubilized membrane fractions derived from *B. subtilis* sporulating cells at hour 2.5 of sporulation. (A) Immunoprecipitates using anti-SpoIIIAE antibody resin from a spoIIIAE+ strain (wt, BDR94) and a δspoIIIAE mutant (δE, BDT2535) are shown. (B) Immunoprecipitates using anti-SpoIIIAF antibody resin from a spoIIIAF+ strain (wt, BDR94) and a δ*spoIIIAF* mutant (δF, BDT2537) are shown. The detergent-solubilized membrane fraction prior to immunoprecipitation (Load), the supernatants after immunoprecipitation (Sup), and the immunoprecipitates (IP) were subjected to immunoblot analysis using anti-SpoIIIAE (E), anti-SpoIIIAF (F), and anti-SpoIIIAG (G) antibodies. All four strains contained a δspoIVB mutation to prevent cleavage of proteins that have domains that reside in the intermembrane space (K. Marquis, N. Campo, TD, and DZR, unpublished observations).
(0.26 MB TIF)

Figure S4 SpoIIIAG resides in a membrane complex with SpoIIIAH and SpoIIQ. Immunoprecipitations were performed with cleared lysates from sporulating *B. subtilis*. Cells were collected at hour 2.5 and treated with lysozyme. Protoplasts were lysed in a hypotonic buffer (50 mM Tris pH 7.5, 1 mM EDTA). Lysates were centrifuged (at 10,000×g for 5 minutes) to remove cellular debris. Cleared lysates were subjected to immunoprecipitation with anti-FLAG M2-agarose (σ). (A) Immunoprecipitations with cells expressing FLAG-tagged SpoIIIAH. Lysates were incubated in the presence or absence of 1% TritonX-100. Cleared lysates (Load), the supernatants after immunoprecipitation (Sup), and the immunoprecipitates (IP) were subjected to immunoblot analysis using anti-FLAG to detect SpoIIIAH (H), anti-SpoIIIAG (G), anti-SpoIIQ (IIQ), and anti-SpoIID (IID) antibodies. (B) Immunoprecipitation with cells expressing FLAG-tagged FtsH. Cleared lysates (Load), the supernatants after immunoprecipitation (Sup), and the immunoprecipitates (IP) were subjected to immunoblot analysis using anti-FLAG to detect FtsH (FtsH) and anti-SpoIIIAG (G) antibodies.
(0.38 MB TIF)

Figure S5 Morphological defects in the absence of SpoIIIA proteins. Forespore morphology was monitored by fluorescence microscopy at hour 3 of sporulation in a wild-type background (wt, BCM703), a Δ*spoIIIAB* mutant (δB, BCM704), a δ*spoIIIAE* mutant (δE, BTD3062), and a strain lacking σ^G^ (δsigG, BCM708). All strains contained a forespore reporter (P*spoIIQ-cfp*; false-colored blue in the lower panel) to visualize the forespore cytoplasm and a YFP-SpoIVFA fusion (IVFA; false-colored green in the lower panel) that labels the mother cell membranes that surround the forespore. The membranes (mb) from the same field were visualized with the fluorescent dye TMA-DPH (false-colored red in the lower panel). Scale bar, 1 µm.
(1.94 MB TIF)

Figure S6 Low levels of SpoIIIAE are sufficient to maintain proper forespore morphology. Forespore morphology was monitored by fluorescence microscopy at hour 3 of sporulation in a wild-type background (wt, BCM703), a δ*spoIIIAE* mutant (δE, BTD3062), a δ*spoIIIAE* mutant containing low levels of SpoIIIAE (low E, BTD3063), and a δ*spoIIIAE* mutant containing high levels of SpoIIIAE (high E, BTD3064). All strains contained a forespore reporter (PspoIIQ-cfp; false-colored blue in the lower panel) to visualize the forespore cytoplasm and a YFP-SpoIVFA fusion (IVFA; false-colored green in the lower panel) that labels the mother cell membranes that surround the forespore. The membranes (mb) from the same field were visualized with the fluorescent dye TMA-DPH (false-colored red in the lower panel). Carets highlight examples of “collapsed” forespores. Scale bar, 1 µm.
(2.12 MB TIF)

Figure S7 σ^G^ is active when synthesized prior to the completion of engulfment. (A) Larger fields of cells showing premature synthesis of σ^G^ result in early σ^G^ activity. σ^G^ activity was assessed by microscopy using a fluorescence reporter (PsspE-gfp) in a δsigG mutant (δsigG, BTD3004), a wild-type background (wt, BTD3002), a δsigG mutant containing one copy of PspoIIQ-sigG (PIIQ-sigG, BTD3007), three copies of PspoIIQ-sigG (3× PIIQ-sigG, BCM791), a δsigG, δ*spoIIIAE* double mutant that contains three copies of PspoIIQ-sigG (δE, BCM816), and a δsigG, δspoIIQ double mutant that contains three copies of *PspoIIQ-sigG* (δIIQ, BCM814). Sporulating cells were monitored at hour 2 of sporulation. The membranes from the same field were visualized using the dye TMA-DPH (false-colored red) and merged with the GFP signal (false-colored green). (B) Late σ^G^ activity requires SpoIIIA and SpoIIQ proteins. σ^G^ activity was quantified at hour 3.5 of sporulation from the same strains as above. The total fluorescence intensity of GFP was measured in each forespore from one field (>400 forespores per strain). Background fluorescence from the same measured region was subtracted. The histogram shows the distribution of GFP intensity in BCM791, BCM814, BCM816.
(3.05 MB TIF)

Figure S8 Early σ^G^ activity is higher and more prevalent in the absence of CsfB/Gin. σ^G^ activity was assessed in single cells by microscopy using a fluorescent reporter (PsspE-gfp) in a wild-type background (wt, BTD3002), in a δsigG mutant (δsigG, BTD3095), a δsigG mutant containing three copies of PspoIIQ-sigG (3× PIIQ-sigG, BTD3100), and the same strain lacking Gin/CsfB (3× PIIQ-sigG, Δgin, BTD3102). Sporulating cells were monitored at hour 2 of sporulation. The membranes from the same field were visualized using the dye TMA-DPH (false-colored red) and merged with the GFP signal (false-colored green). The membrane dye inefficiently traverses the lipid bilayer and therefore reports on the engulfment status of the forespore [1]. Forespores that stain weakly with TMA-DPH have been completely engulfed by the mother cell. Forespores that have not yet completed engulfment have strong signal due to the two membranes surrounding the spore. Yellow carets highlight examples of forespore that have σG activity but have not completed engulfment. The fluorescence intensities of the GFP reporter in the δgin strain are ∼2-fold higher than the intensities in the matched control strain. Scale bar, 1 µm.
(1.93 MB TIF)

Figure S9 A subset of cells that are blocked for engulfment have σG activity. (A) σG activity was assessed in single cells by microscopy using a fluorescent reporter (PsspE-gfp) in a wild-type background (wt, BTD3002), a δspoIID mutant (δIID, BTD3085), and a δspoIID, spoIIIA(D224A) double mutant (δIID, A(D224A), BTD3086). Cells were visualized at hour 3 of sporulation. Forespore GFP fluorescence (false-colored green in the lower panel) and the fluorescent membrane dye TMA-DPH (false-colored red in the lower panel) are shown. (B) Large fields of sporulating cells from the same three strains showing forespore CFP fluorescence. The percentage of sporulating cells that have σG activity is shown below the field.
(2.16 MB TIF)

Table S1 Sporulation efficiency of the SpoIIIA mutants. Sporulation efficiency was determined in strains harboring in-frame deletions of *spoIIIAA* (A) through *spoIIIAH* (H) and isogenic strains expressing the corresponding *spoIIIA* gene inserted at a non-essential locus.
(0.11 MB TIF)

Table S2 Strains used in this study.
(0.07 MB DOC)

Table S3 Plasmids used in this study.
(0.04 MB DOC)

Table S4 Oligonucleotide primers used in this study.
(0.03 MB DOC)

Text S1 Supplemental materials and methods.
(0.06 MB DOC)
