# Supplementary figures and images for: Novel Secretion Apparatus Maintains Spore Integrity and Developmental Gene Expression in Bacillus subtilis
Source: PLoS Genet. 2009 Jul 17;5(7):e1000566. doi: 10.1371/journal.pgen.1000566 (PMC2703783; doi:10.1371/journal.pgen.1000566)

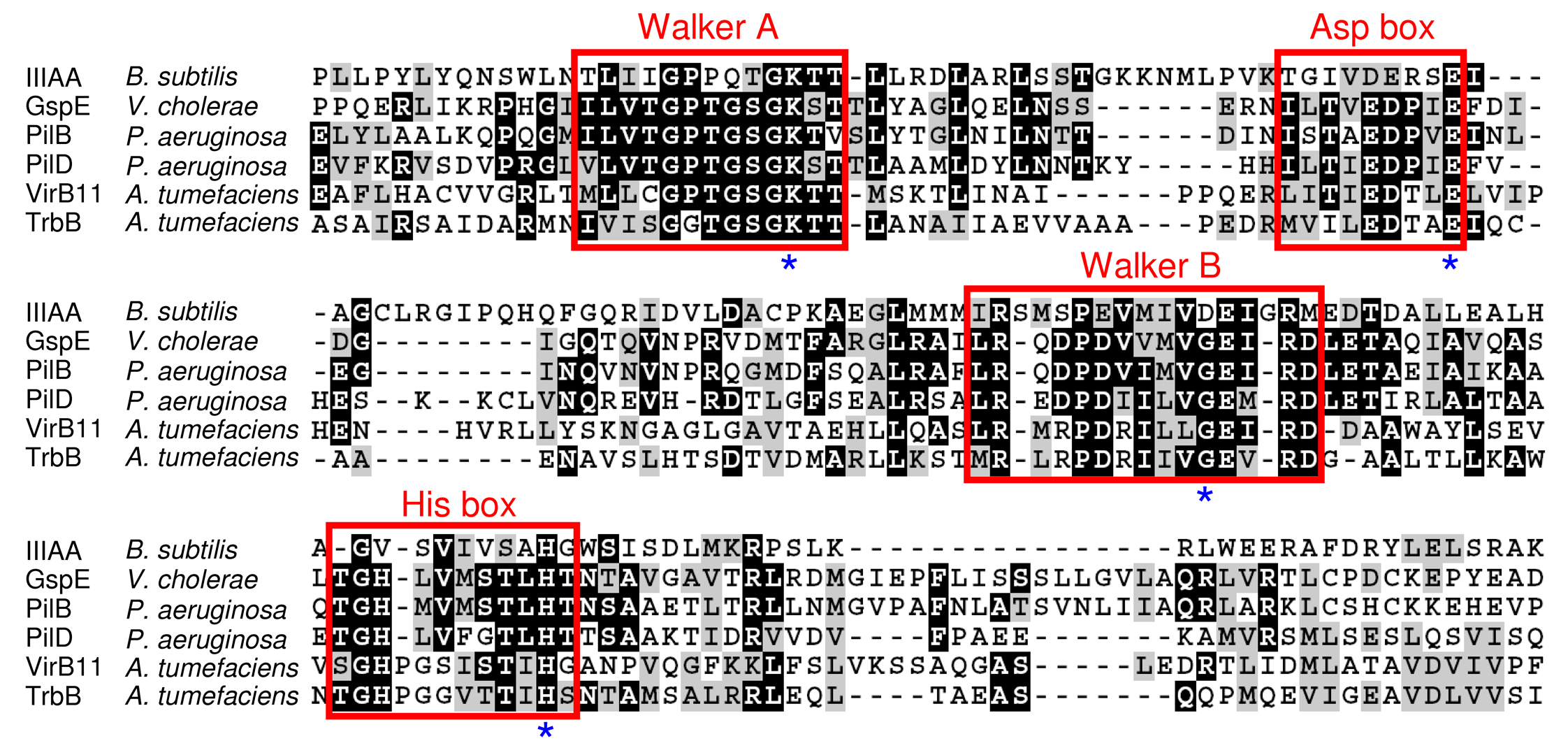

Supplement: Figure S1 — Sequence alignment of the C-terminal domain of SpoIIIAA (IIIAA) from B. subtilis with the ATPase domains of several secretion ATPases. The conserved motifs (red boxes) found in all secretion NTPases are highlighted. Mutated residues are indicated (blue asterisk). The alignment was made using ClustalW (http://www.ch.embnet.org/software/ClustalW.html) and BOXSHADE (http://www.ch.embnet.org/software/BOX_form.html). (0.96 MB TIF) [file pgen.1000566.s001.tif]

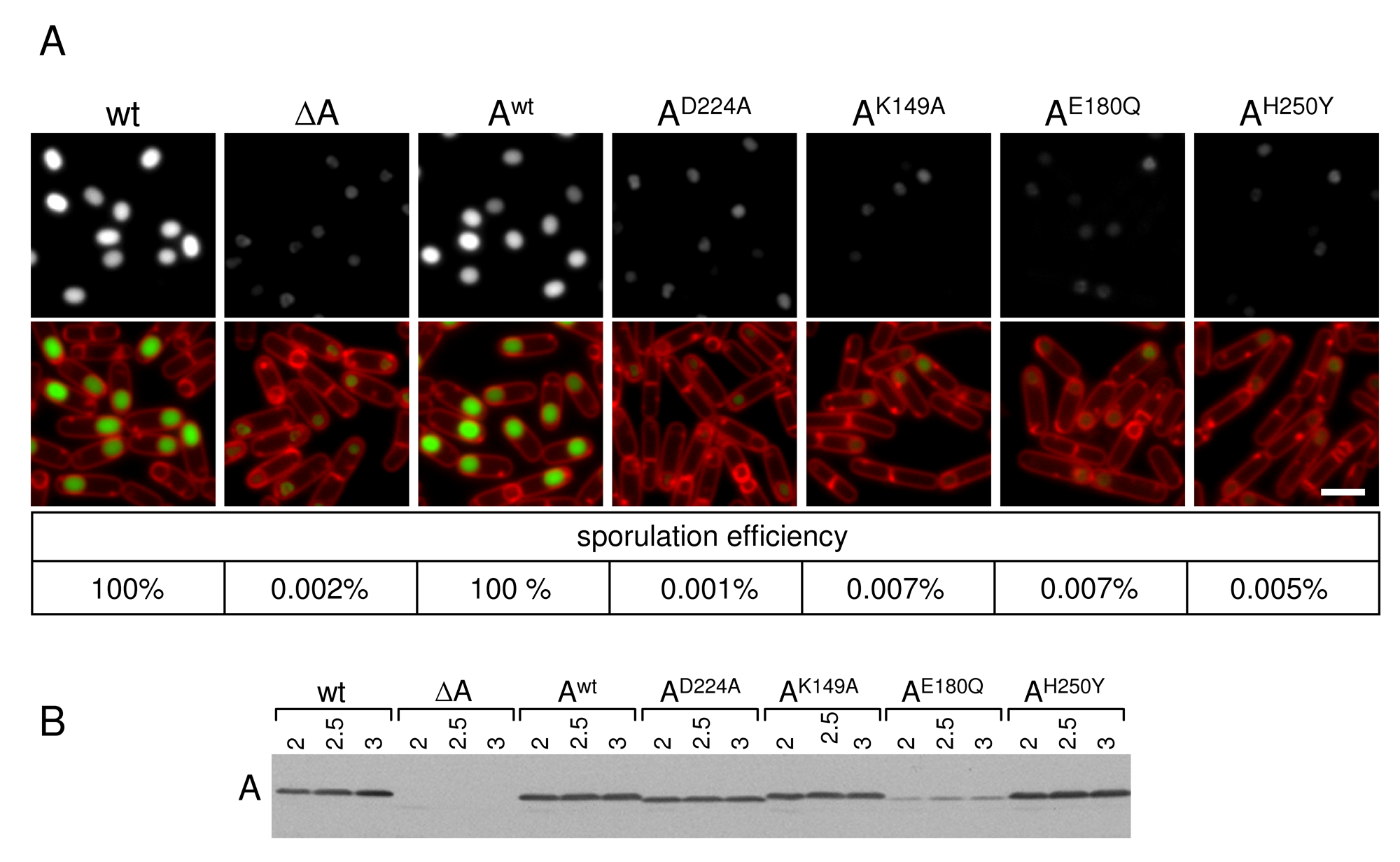

Supplement: Figure S2 — The ATPase motifs in SpoIIIAA are required for σG activity and sporulation efficiency. (A) σG activity was assessed in single cells by microscopy using a fluorescent reporter (PsspE-cfp) in a wild-type background (wt, BTD1609), a δspoIIIAA mutant (δA, BTD2713), a δspoIIIAA mutant containing a wild-type copy of spoIIIAA inserted at a non-essential locus (A(wt), BTD2719), a spoIIIAA D224A Walker B box point mutant (AD224A, BTD2775), a spoIIIAA K149A Walker A box point mutant (AK149A, BTD2906), a spoIIIAA E180Q Asp box point mutant (AE180Q, BTD2907), and a spoIIIAA H250Y His box point mutant (AH250Y, BTD2909). Cells were visualized at hour 3 of sporulation. Forespore CFP fluorescence (false-colored green in the lower panel) and the fluorescent membrane dye TMA-DPH (false-colored red) are shown. Scale bar, 1 µm. Sporulation efficiencies of the same strains are shown below the fluorescent images. (B) Immunoblot analysis of whole cell lysates from sporulating cells shown in A. Time (in hours) after the initiation of sporulation is indicated. (0.78 MB TIF) [file pgen.1000566.s002.tif]

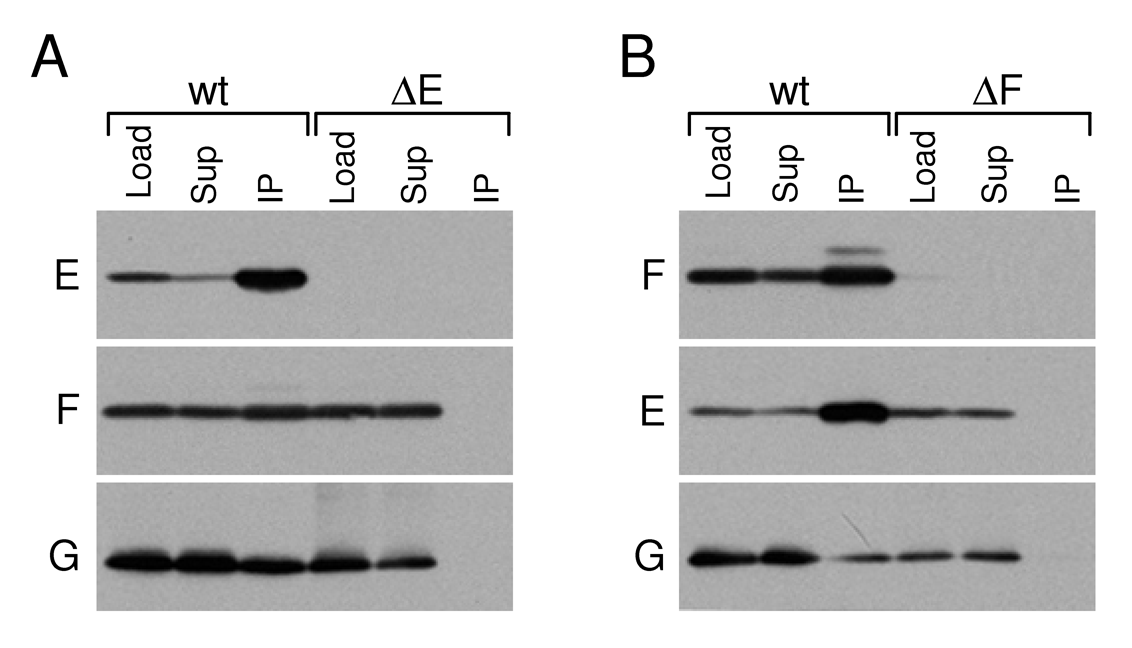

Supplement: Figure S3 — The SpoIIIAE, SpoIIIAF and SpoIIIAG proteins reside in a membrane complex. Immunoprecipitations were performed on detergent-solubilized membrane fractions derived from B. subtilis sporulating cells at hour 2.5 of sporulation. (A) Immunoprecipitates using anti-SpoIIIAE antibody resin from a spoIIIAE+ strain (wt, BDR94) and a δspoIIIAE mutant (δE, BDT2535) are shown. (B) Immunoprecipitates using anti-SpoIIIAF antibody resin from a spoIIIAF+ strain (wt, BDR94) and a δspoIIIAF mutant (δF, BDT2537) are shown. The detergent-solubilized membrane fraction prior to immunoprecipitation (Load), the supernatants after immunoprecipitation (Sup), and the immunoprecipitates (IP) were subjected to immunoblot analysis using anti-SpoIIIAE (E), anti-SpoIIIAF (F), and anti-SpoIIIAG (G) antibodies. All four strains contained a δspoIVB mutation to prevent cleavage of proteins that have domains that reside in the intermembrane space (K. Marquis, N. Campo, TD, and DZR, unpublished observations). (0.26 MB TIF) [file pgen.1000566.s003.tif]

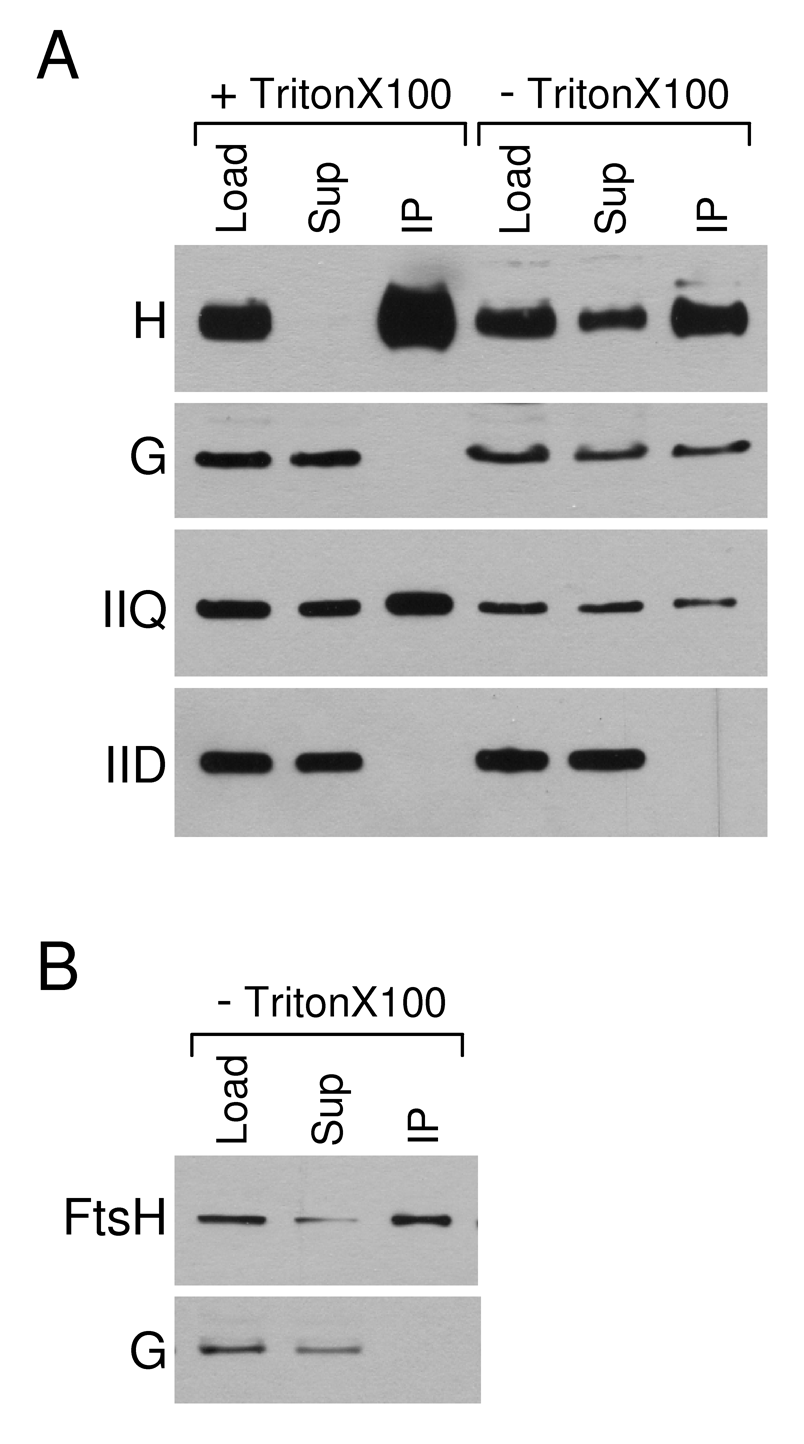

Supplement: Figure S4 — SpoIIIAG resides in a membrane complex with SpoIIIAH and SpoIIQ. Immunoprecipitations were performed with cleared lysates from sporulating B. subtilis. Cells were collected at hour 2.5 and treated with lysozyme. Protoplasts were lysed in a hypotonic buffer (50 mM Tris pH 7.5, 1 mM EDTA). Lysates were centrifuged (at 10,000×g for 5 minutes) to remove cellular debris. Cleared lysates were subjected to immunoprecipitation with anti-FLAG M2-agarose (σ). (A) Immunoprecipitations with cells expressing FLAG-tagged SpoIIIAH. Lysates were incubated in the presence or absence of 1% TritonX-100. Cleared lysates (Load), the supernatants after immunoprecipitation (Sup), and the immunoprecipitates (IP) were subjected to immunoblot analysis using anti-FLAG to detect SpoIIIAH (H), anti-SpoIIIAG (G), anti-SpoIIQ (IIQ), and anti-SpoIID (IID) antibodies. (B) Immunoprecipitation with cells expressing FLAG-tagged FtsH. Cleared lysates (Load), the supernatants after immunoprecipitation (Sup), and the immunoprecipitates (IP) were subjected to immunoblot analysis using anti-FLAG to detect FtsH (FtsH) and anti-SpoIIIAG (G) antibodies. (0.38 MB TIF) [file pgen.1000566.s004.tif]

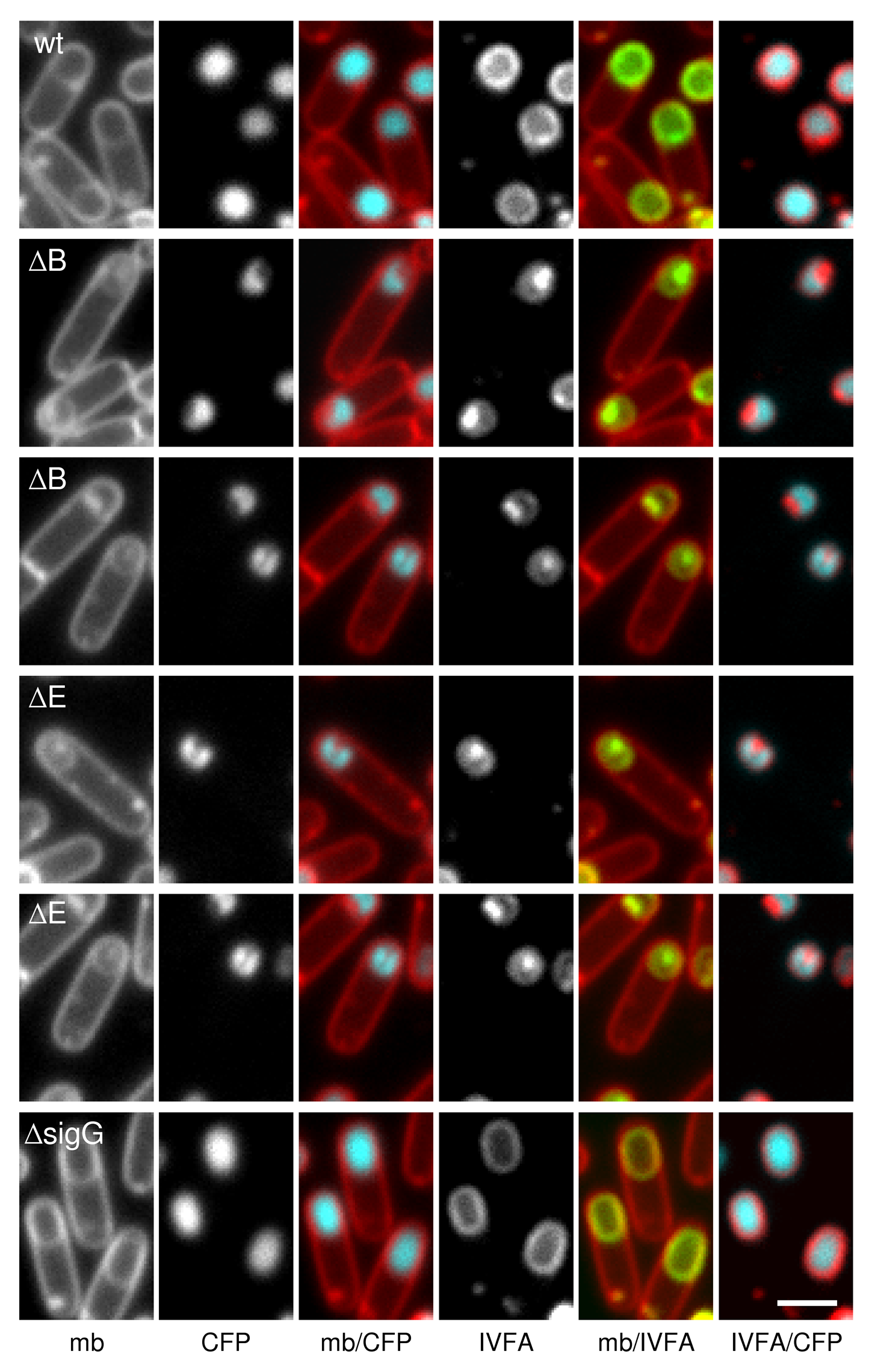

Supplement: Figure S5 — Morphological defects in the absence of SpoIIIA proteins. Forespore morphology was monitored by fluorescence microscopy at hour 3 of sporulation in a wild-type background (wt, BCM703), a ΔspoIIIAB mutant (δB, BCM704), a δspoIIIAE mutant (δE, BTD3062), and a strain lacking σG (δsigG, BCM708). All strains contained a forespore reporter (PspoIIQ-cfp; false-colored blue in the lower panel) to visualize the forespore cytoplasm and a YFP-SpoIVFA fusion (IVFA; false-colored green in the lower panel) that labels the mother cell membranes that surround the forespore. The membranes (mb) from the same field were visualized with the fluorescent dye TMA-DPH (false-colored red in the lower panel). Scale bar, 1 µm. (1.94 MB TIF) [file pgen.1000566.s005.tif]

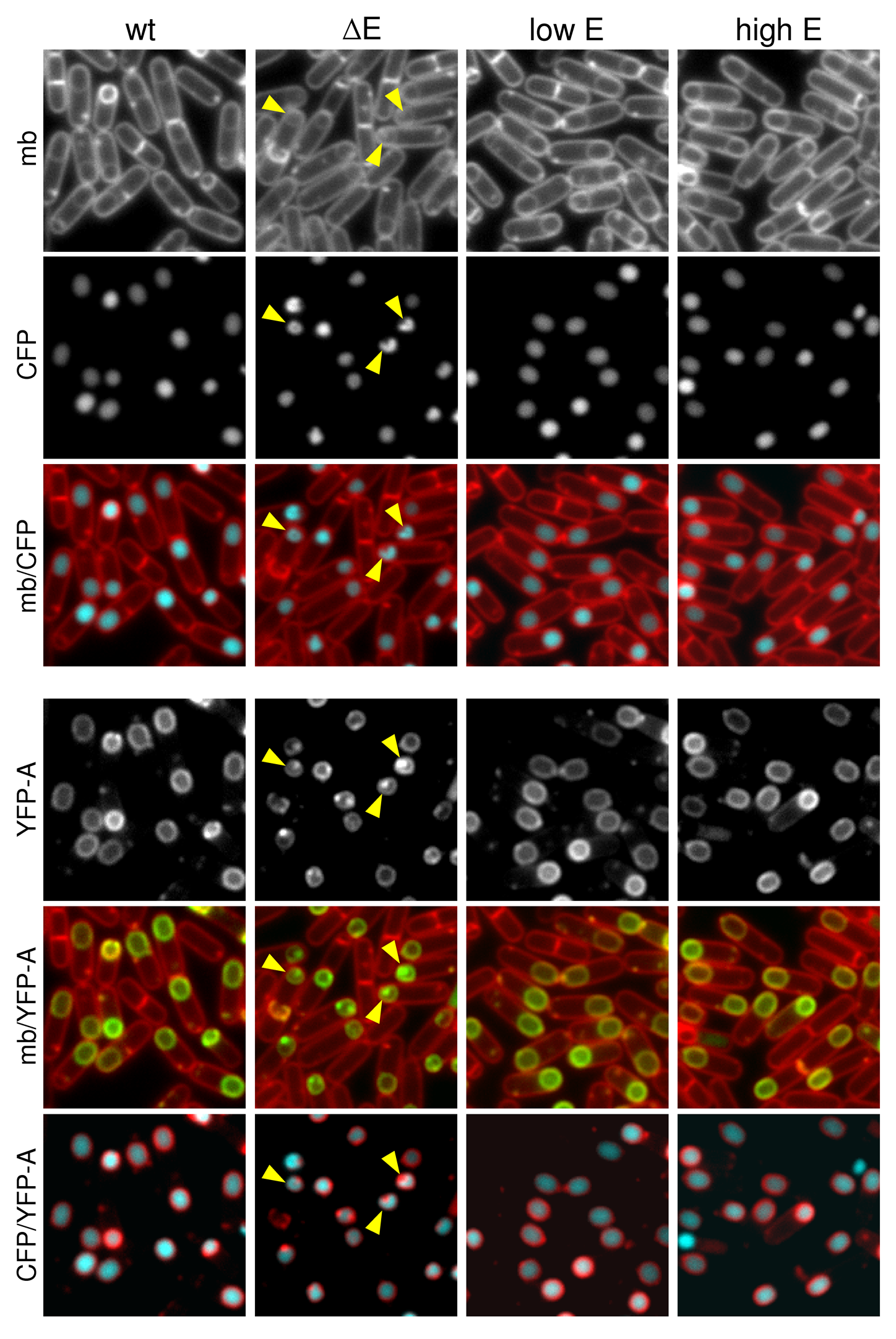

Supplement: Figure S6 — Low levels of SpoIIIAE are sufficient to maintain proper forespore morphology. Forespore morphology was monitored by fluorescence microscopy at hour 3 of sporulation in a wild-type background (wt, BCM703), a δspoIIIAE mutant (δE, BTD3062), a δspoIIIAE mutant containing low levels of SpoIIIAE (low E, BTD3063), and a δspoIIIAE mutant containing high levels of SpoIIIAE (high E, BTD3064). All strains contained a forespore reporter (PspoIIQ-cfp; false-colored blue in the lower panel) to visualize the forespore cytoplasm and a YFP-SpoIVFA fusion (IVFA; false-colored green in the lower panel) that labels the mother cell membranes that surround the forespore. The membranes (mb) from the same field were visualized with the fluorescent dye TMA-DPH (false-colored red in the lower panel). Carets highlight examples of “collapsed” forespores. Scale bar, 1 µm. (2.12 MB TIF) [file pgen.1000566.s006.tif]

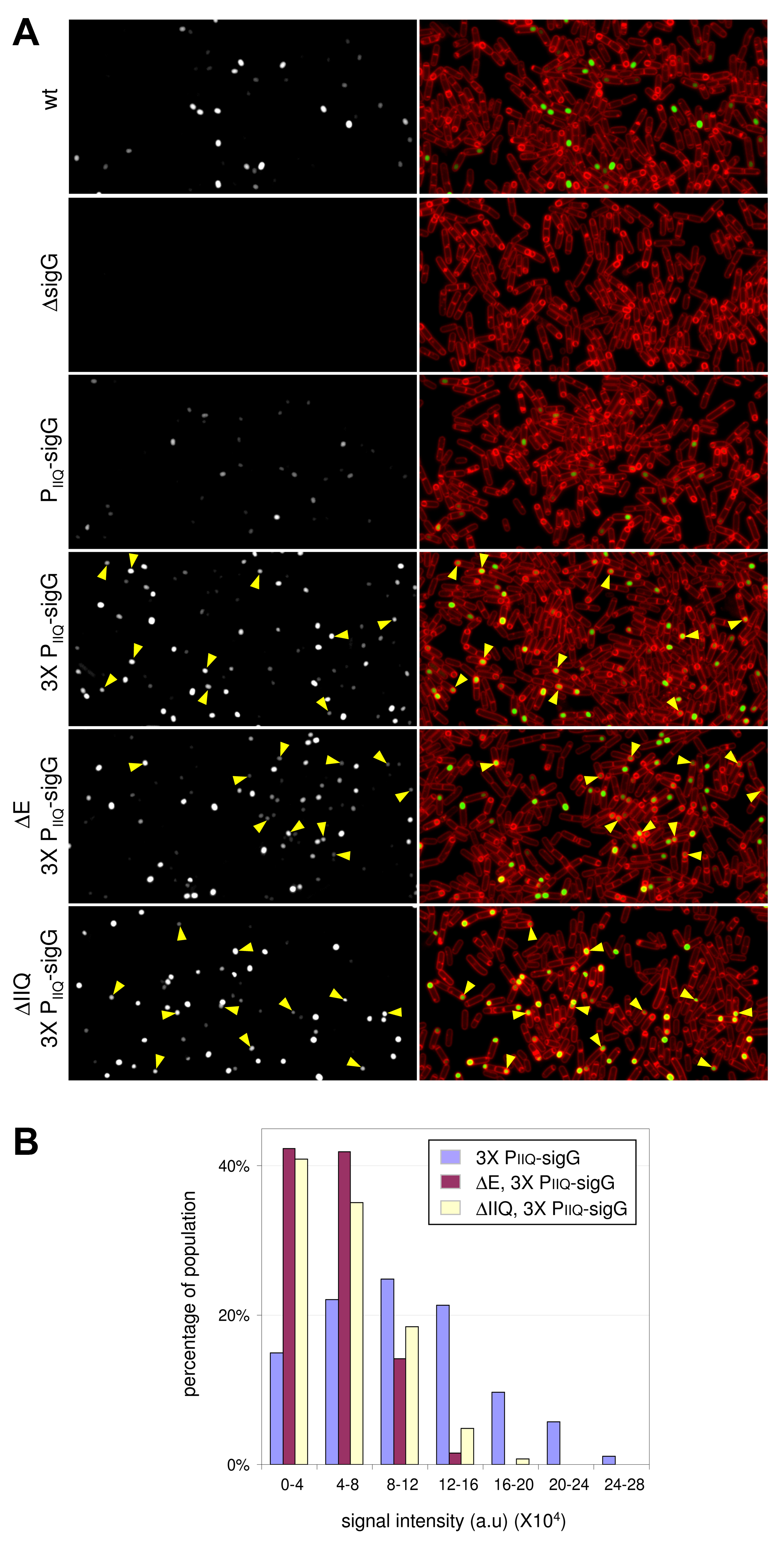

Supplement: Figure S7 — σG is active when synthesized prior to the completion of engulfment. (A) Larger fields of cells showing premature synthesis of σG result in early σG activity. σG activity was assessed by microscopy using a fluorescence reporter (PsspE-gfp) in a δsigG mutant (δsigG, BTD3004), a wild-type background (wt, BTD3002), a δsigG mutant containing one copy of PspoIIQ-sigG (PIIQ-sigG, BTD3007), three copies of PspoIIQ-sigG (3× PIIQ-sigG, BCM791), a δsigG, δspoIIIAE double mutant that contains three copies of PspoIIQ-sigG (δE, BCM816), and a δsigG, δspoIIQ double mutant that contains three copies of PspoIIQ-sigG (δIIQ, BCM814). Sporulating cells were monitored at hour 2 of sporulation. The membranes from the same field were visualized using the dye TMA-DPH (false-colored red) and merged with the GFP signal (false-colored green). (B) Late σG activity requires SpoIIIA and SpoIIQ proteins. σG activity was quantified at hour 3.5 of sporulation from the same strains as above. The total fluorescence intensity of GFP was measured in each forespore from one field (>400 forespores per strain). Background fluorescence from the same measured region was subtracted. The histogram shows the distribution of GFP intensity in BCM791, BCM814, BCM816. (3.05 MB TIF) [file pgen.1000566.s007.tif]

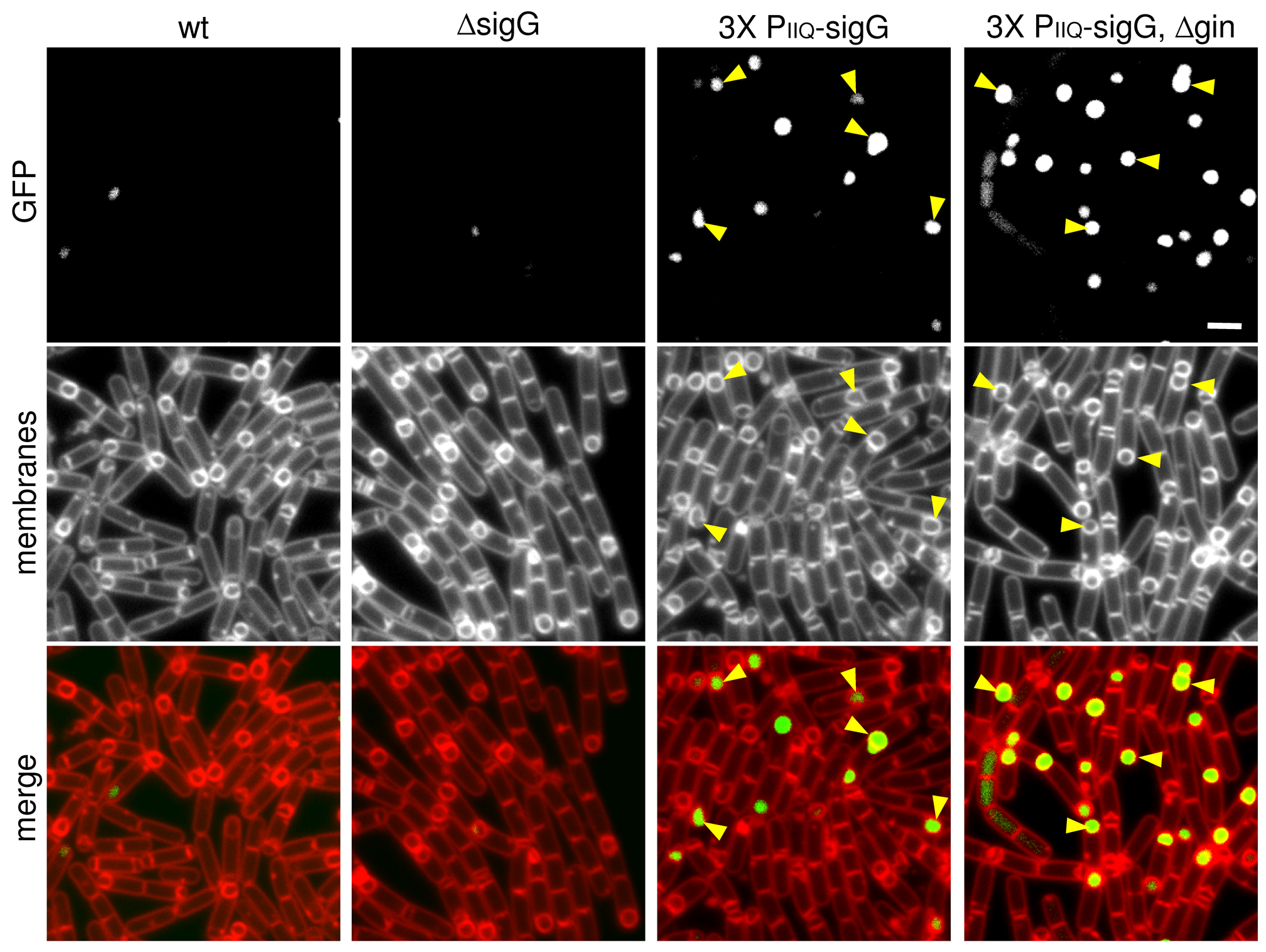

Supplement: Figure S8 — Early σG activity is higher and more prevalent in the absence of CsfB/Gin. σG activity was assessed in single cells by microscopy using a fluorescent reporter (PsspE-gfp) in a wild-type background (wt, BTD3002), in a δsigG mutant (δsigG, BTD3095), a δsigG mutant containing three copies of PspoIIQ-sigG (3× PIIQ-sigG, BTD3100), and the same strain lacking Gin/CsfB (3× PIIQ-sigG, Δgin, BTD3102). Sporulating cells were monitored at hour 2 of sporulation. The membranes from the same field were visualized using the dye TMA-DPH (false-colored red) and merged with the GFP signal (false-colored green). The membrane dye inefficiently traverses the lipid bilayer and therefore reports on the engulfment status of the forespore [1]. Forespores that stain weakly with TMA-DPH have been completely engulfed by the mother cell. Forespores that have not yet completed engulfment have strong signal due to the two membranes surrounding the spore. Yellow carets highlight examples of forespore that have σG activity but have not completed engulfment. The fluorescence intensities of the GFP reporter in the δgin strain are ∼2-fold higher than the intensities in the matched control strain. Scale bar, 1 µm. (1.93 MB TIF) [file pgen.1000566.s008.tif]

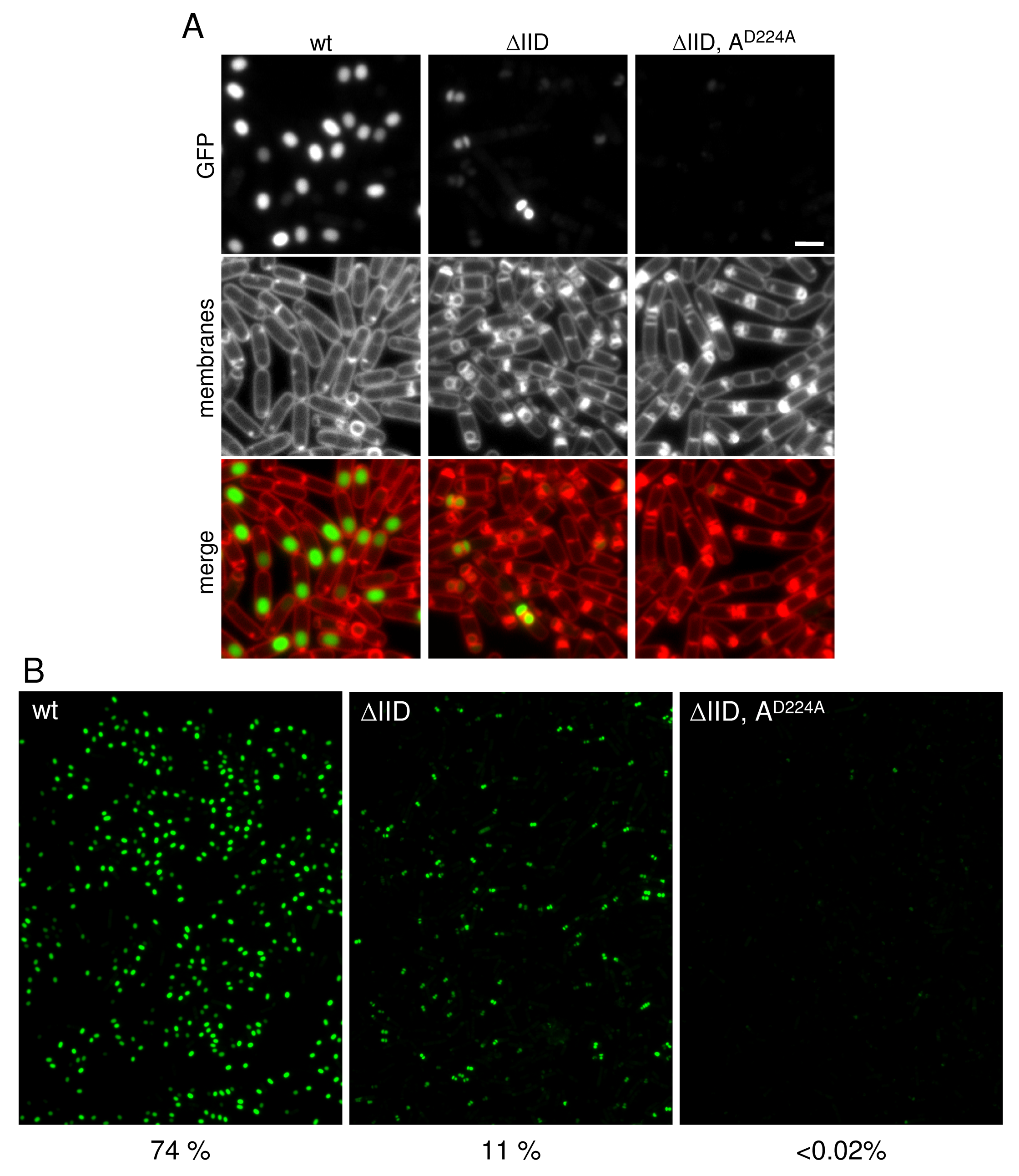

Supplement: Figure S9 — A subset of cells that are blocked for engulfment have σG activity. (A) σG activity was assessed in single cells by microscopy using a fluorescent reporter (PsspE-gfp) in a wild-type background (wt, BTD3002), a δspoIID mutant (δIID, BTD3085), and a δspoIID, spoIIIA(D224A) double mutant (δIID, A(D224A), BTD3086). Cells were visualized at hour 3 of sporulation. Forespore GFP fluorescence (false-colored green in the lower panel) and the fluorescent membrane dye TMA-DPH (false-colored red in the lower panel) are shown. (B) Large fields of sporulating cells from the same three strains showing forespore CFP fluorescence. The percentage of sporulating cells that have σG activity is shown below the field. (2.16 MB TIF) [file pgen.1000566.s009.tif]

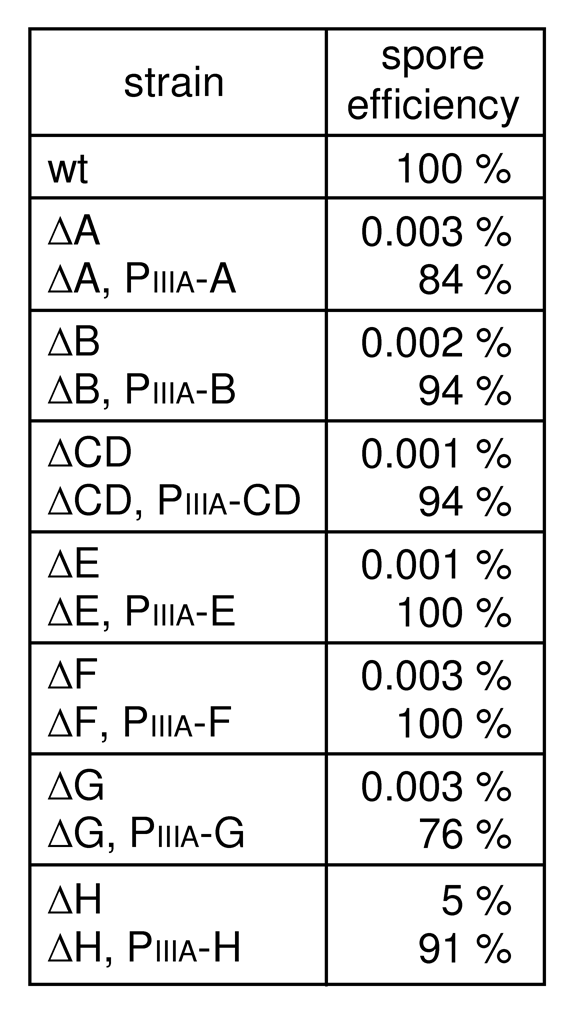

Supplement: Table S1 — Sporulation efficiency of the SpoIIIA mutants. Sporulation efficiency was determined in strains harboring in-frame deletions of spoIIIAA (A) through spoIIIAH (H) and isogenic strains expressing the corresponding spoIIIA gene inserted at a non-essential locus. (0.11 MB TIF) [file pgen.1000566.s010.tif]
