# Supplementary material for: Novel Secretion Apparatus Maintains Spore Integrity and Developmental Gene Expression in Bacillus subtilis
Source: PLoS Genet. 2009 Jul 17;5(7):e1000566. doi: 10.1371/journal.pgen.1000566 (PMC2703783; doi:10.1371/journal.pgen.1000566)
Supplement: Table S2 — Strains used in this study. (0.07 MB DOC) [file pgen.1000566.s011.doc]

**Supplemental Table 2.** *Strains used in this study*

| **Strain** | **Genotype** | **Source** |
| --- | --- | --- |
| PY79 | Prototrophic wild-type strain | Youngman *et al.*, 1983 |
| BTD117 | ∆*spoIIIAA*Ω*pDT21 (phleo)* | This work |
| BTD119 | ∆*spoIIIAB*Ω*pDT21 (phleo)* | This work |
| BTD121 | ∆*spoIIIACD*Ω*pDT21 (phleo)* | This work |
| BTD113 | ∆*spoIIIAE*Ω*pDT21 (phleo)* | This work |
| BTD115 | ∆*spoIIIAF*Ω*pDT21 (phleo)* | This work |
| BTD123 | ∆*spoIIIAG*Ω*pDT21 (phleo)* | This work |
| BTD125 | ∆*spoIIIAH*Ω*pDT21 (phleo)* | This work |
| BTD2341 | ∆*spoIIIAA*Ω*pDT21 (phleo), ycgO::PspoIIIA-RBSspoIIIAA-spoIIIAA (erm)* | This work |
| BTD2345 | ∆*spoIIIAB*Ω*pDT21 (phleo), ycgO::PspoIIIA-RBSspoIIIAA-spoIIIAB (erm)* | This work |
| BTD2371 | ∆*spoIIIACD*Ω*pDT21 (phleo), ycgO::PspoIIIA-RBSspoIIIAA-spoIIIACD (erm)* | This work |
| BTD2349 | ∆*spoIIIAE*Ω*pDT21 (phleo), ycgO::PspoIIIA-RBSspoIIIAA-spoIIIAE (erm)* | This work |
| BCM75 | ∆*spoIIIAF*Ω*pDT21 (phleo), ycgO::PspoIIIA-RBSspoIIIAA-spoIIIAF (erm)* | This work |
| BTD2353 | ∆*spoIIIAG*Ω*pDT21 (phleo), ycgO::PspoIIIA-RBSspoIIIAA-spoIIIAG (erm)* | This work |
| BTD2357 | ∆*spoIIIAH*Ω*pDT21 (phleo), ycgO::PspoIIIA-RBSspoIIIAA-spoIIIAH (erm)* | This work |
| BTD2713 | *yycR::PsspE-RBSopt-cfp (cat),* ∆*spoIIIAA*Ω*pDT21 (phleo),* | This work |
| BTD2775 | *yycR::PsspE-RBSopt-cfp (cat),* ∆*spoIIIAA*Ω*pDT21 (phleo), amyE::PspoIIIA-RBSIIIAA-spoIIIAA^D224A^ (spec)* | This work |
| BTD2779 | *yycR::PsspE-RBSopt-cfp (cat),* ∆*spoIIIAA*Ω*pDT21 (phleo), amyE::PspoIIIA-RBSIIIAA-spoIIIAA^wt^ (spec)* | This work |
| BTD1331 | *sspB-lacZ (cat), sigG::neo* | This work |
| BTD2917 | *sspB-lacZ (cat),* ∆*spoIIIAA*Ω*pDT21 (phleo)* | This work |
| BTD2919 | *sspB-lacZ (cat),* ∆*spoIIIAA*Ω*pDT21 (phleo), amyE::PspoIIIA-RBSIIIAA-spoIIIAA^wt^ (spec),* | This work |
| BTD2920 | *sspB-lacZ (cat),* ∆*spoIIIAA*Ω*pDT21 (phleo), amyE::PspoIIIA-RBSIIIAA-spoIIIAA^D224A^ (spec)* | This work |
| BTD2867 | *spoIVB::cat, amyE::PspoIIIA-RBSA-myc3-spoIIIAD (spec),* ∆*spoIIIAG*Ω*pDT21 (phleo)* | This work |
| BTD2869 | *spoIVB::cat, amyE::PspoIIIA-RBSA-myc3-spoIIIAD (spec)* | This work |
| BTD665 | *spoIIQ::spec, spoIVB::erm, sacA::PspoIIQ-RBSopt-cfp-spoIIQ (tet)* | Campo *et al.*, 2008 |
| BDR94 | *spoIVB::spec* | Doan and Rudner, 2007 |
| BTD49 | *amyE::PspoIIIA-RBSspoIIIAA-cfp-spoIIIAG (spec)* | This work |
| BCM575 | *amyE::PspoIIIA-RBSspoIIIAA-cfp-spoIIIAG (spec),* ∆*spoIIIAH*Ω*pDT21 (phleo)* | This work |
| BCM736 | *amyE::PspoIIIA-RBSspoIIIAA-cfp-spoIIIAG (spec),spoIIQ::cat* | This work |
| BCM703 | *yvbJ::PspoIIQ-RBSopt-cfp (spec), lacA::PspoIVF-RBSopt-yfp-spoIVFA (erm)* | This work |
| BCM704 | *yvbJ::PspoIIQ-RBSopt-cfp (spec), lacA::PspoIVF-RBSopt-yfp-spoIVFA (erm),* ∆*spoIIIAB*Ω*pDT21 (phleo)* | This work |
| BCM706 | *yvbJ::PspoIIQ-RBSopt-cfp (spec), pelB::PspoIVF-RBSopt-yfp-spoIVFA (cat), spoIIIA::erm* | This work |
| BCM708 | *yvbJ::PspoIIQ-RBSopt-cfp (spec), lacA::PspoIVF-RBSopt-yfp-spoIVFA (erm), sigG::neo* | This work |
| BCM716 | *yvbJ::PspoIIQ-RBSopt-cfp (spec), lacA::PspoIVF-RBSopt-yfp-spoIVFA (erm), spoIIQ::cat* | This work |
| BCM810 | *amyE::PspoIIIA-RBSspoIIIAA-cfp-spoIIIAG (spec),* ∆*spoIIIAE*Ω*pDT21 (phleo)* | This work |
| BDR841 | *spoIIIA::erm* | This work |
| BTD141 | *spoIIQ::cat* | This work |
| BDR104 | *sigG::neo* | Margolis P. *et al.*, 1991 |
| BTD2683 | ∆*spoIIIAA*Ω*pDT21 (phleo), amyE::PspoIIIA-RBSspoIIIAA-spoIIIAA^D224A^ (cat)* | This work |
| BTD3019 | ∆*spoIIIAE*Ω*pDT21 (phleo), ycgO::PspoIIIA-RBSspoIVFA-ttg-spoIIIAE (erm)* | This work |
| BTD3023 | ∆*spoIIIAA*Ω*pDT21 (phleo), ycgO::PspoIIIA-RBSspoIVFA-ttg-spoIIIAA (erm)* | This work |
| BTD3002 | *ycgO::PsspE-RBSopt-gfp (spec)* | This work |
| BTD3004 | *ycgO::PsspE-RBSopt-gfp (spec), sigG::neo* | This work |
| BTD3007 | *ycgO::PsspE-RBSopt-gfp (spec), sigG::neo, amyE::PspoIIQ-sigG (cat)* | This work |
| BCM791 | *ycgO::PsspE-RBSopt-gfp (spec), sigG::neo, amyE::PspoIIQ-sigG (cat), lacA::PspoIIQ-sigG (tet), yhdG::PspoIIQ-sigG (tet)* | This work |
| BCM816 | *ycgO::PsspE-RBSopt-gfp (spec), sigG::neo, amyE::PspoIIQ-sigG (cat), lacA::PspoIIQ-sigG (tet), yhdG::PspoIIQ-sigG (tet),* ∆*spoIIIAE*Ω*pDT21 (phleo)* | This work |
|  |  |  |
| MO1194 | Δ*spoIIIAA, trpC2, pheA1* | Gift from P. Stragier |
| MO1195 | Δ*spoIIIAB, trpC2, pheA1* | Gift from P. Stragier |
| MO1227 | Δ*spoIIIACD, trpC2, pheA1* | Gift from P. Stragier |
| MO1179 | Δ*spoIIIAE, trpC2, pheA1* | Gift from P. Stragier |
| MO1180 | Δ*spoIIIAF, trpC2, pheA1* | Gift from P. Stragier |
| MO1422 | Δ*spoIIIAG, trpC2, pheA1* | Gift from P. Stragier |
| MO1429 | Δ*spoIIIAH, trpC2, pheA1* | Gift from P. Stragier |
| PM8 | *sspB-lacZ (cat)* | Mason *et al.*, 1988 |
| MO2021 | *spoIIQ::spec, trpC2, phe A1* | Londono-Vallejo *et al.*, 1997 |
| MO3196 | *sigG::erm, amyE::PspoIIQ-sigG (cat), trpC2, pheA1* | Gift from P. Stragier |
| MO1433 | *spoIIIA::erm, trpC2, pheA1* | Gift from P. Stragier |
|  |  |  |
|  |  |  |
|  |  |  |
|  |  |  |
|  |  |  |
|  |  |  |
