# Supplementary material for: Novel Secretion Apparatus Maintains Spore Integrity and Developmental Gene Expression in Bacillus subtilis
Source: PLoS Genet. 2009 Jul 17;5(7):e1000566. doi: 10.1371/journal.pgen.1000566 (PMC2703783; doi:10.1371/journal.pgen.1000566)
Supplement: Table S3 — Plasmids used in this study. (0.04 MB DOC) [file pgen.1000566.s012.doc]

**Supplemental Table 3.** *Plasmids used in this study*

| **plasmid** | **description** | **source** |
| --- | --- | --- |
| pDT19 | *amyE::PspoIIIA-RBSspoIIIAA-cfp-spoIIIAG (spec)* | This work |
| pTD21 | *spoIIIAH^wt^Ωphleo* | This work |
| pDT195 | *ycgO::PspoIIIA-spoIIIAA (erm)* | This work |
| pDT196 | *ycgO::PspoIIIA-spoIIIAB (erm)* | This work |
| pDT199 | *ycgO::PspoIIIA-spoIIIAE (erm)* | This work |
| pDT201 | *ycgO::PspoIIIAspoIIIAG (erm)* | This work |
| pDT202 | *ycgO::PspoIIIA-spoIIIAH (erm)* | This work |
| pDT204 | *ycgO::PspoIIIA-spoIIIACD (erm)* | This work |
| pDT246 | *amyE::PspoIIIA-myc_3_-spoIIIAD (spec)* | This work |
| pDT257 | *amyE::spoIIIAA^D224A^ (cat)* | This work |
| pDT259 | *yycR::PsspE-cfp (cat)* | This work |
| pDT266 | *amyE::spoIIIAA^wt^ (spec)* | This work |
| pDT267 | *amyE::spoIIIAA^D224A^ (spec)* | This work |
| pDT307 | *ycgO::PsspE-gfp (spec)* | This work |
| pDT326 | *ycgO::PspoIIIA-RBSspoIVFA-ttg-spoIIIAE (erm)* | This work |
| pDT330 | *ycgO::PspoIIIA-RBSspoIVFA-ttg-spoIIIAA (erm)* | This work |
| pCM16 | *ycgO::PspoIIIA-spoIIIAF (erm)* | This work |
| pNS38 | *yvbJ::PspoIIQ-cfp (spec)* | Sullivan et al., submitted |
| pKM30 | *lacA::PspoIVF-yfp-spoIVFA (erm)* | Doan et al*.*, 2004 |
| pKM108 | *his_6_-spoIIIAG_(fragment)_* | This work |
| pDT152 | *his_6_-spoIIIAA* | This work |
| pDT170 | *his_6_-spoIIIAF_(fragment)_* | This work |
| pDT178 | *GST-spoIIIAE_(fragment)_* | This work |
