# Supplementary material for: Novel Secretion Apparatus Maintains Spore Integrity and Developmental Gene Expression in Bacillus subtilis
Source: PLoS Genet. 2009 Jul 17;5(7):e1000566. doi: 10.1371/journal.pgen.1000566 (PMC2703783; doi:10.1371/journal.pgen.1000566)
Supplement: Table S4 — Oligonucleotide primers used in this study. (0.03 MB DOC) [file pgen.1000566.s013.doc]

**Supplemental Table 4.** *Oligonucleotide primers used in this study*

| **primer** | **sequence** |
| --- | --- |
| oTD1 | *gccGAATTctgacggcagcaattgtcatgc* |
| oTD2 | *gccGGATCCatactcgagtctaagcttggcttctttaaaatgtatgatgtg* |
| oTD3 | *cgcAAGCTTagaaaggaggaggctctgatggtttcaaaaggcgaagaactg* |
| oTD4 | *gcgCTCGAGcttataaagttcgtccatgcc* |
| oTD6 | *gcgCTCGAGatgaataaaaacggattatggaatg* |
| oTD7 | *gccGGATCCttatgaatcctcctttatttttttagg* |
| oDT8 | *gcgCTCGAGttgaatgaaatcgctgaggttctc* |
| oDT9 | *gccGGATCCtcagcatgttttcacgcccgtcgtcc* |
| oDT11 | *cgcGGATCCttatttagagggttcaaatgtgac* |
| oDT15 | *cgcAAGCTTagaaaggaggaggctctgttgaatgaaatcgctgaggttctc* |
| oDT135 | *ttccatacgcccgatttctgcgacaatcatcacctcagg* |
| oDT136 | *cctgaggtgatgattgtcgcagaaatcgggcgtatggaa* |
| oDT150 | *gccGCTAGCatgaatgaaatcgctgaggttctc* |
| oDT151 | *cggCTCGAGttagcatgttttcacgcccgtcgt* |
| oTD164 | *cggCTCGAGttacacttcatgaaacaaataagaa* |
| oTD169 | *cgcGCTAGCaagacagaccccgaagtc* |
| oTD170 | *cggCTCGAgttattcattgccgacactctc* |
| oDT176 | *gccGGATCCgaaattgtacaagcagctggt* |
| oDT181 | *gccGCTAGCagaaaggaggaggctctgatgctgaagctgctgggc* |
| oDT182 | *gccGGATCCttacatcaatagaagaatgagtaac* |
| oDT184 | *cgcAAGCTTagaaaggaggaggctctgatgggagtagacgtgaatg* |
| oDT188 | *gccGGATCCttatgacatagaaggtataagtcc* |
| oTD190 | *cgcAAGCTTagaaaggaggaggctctgttgaagcgctttcaatgggtt* |
| oTD191 | *gccGGATCCttattgcctgcctccttcattt* |
| oTD196 | *cgcAAGCTTagaaaggaggaggctctgatgaataaaaacggattatgg* |
| oDT198 | *cgcAAGCTTagaaaggaggaggctctgatgcttaaaaaacaaaccgtt* |
| oTD200 | *gccGCTAGCggcttctttaaaatgtatgatg* |
| oTD201 | *gccGGATCCTTAttcattgccgacactctcc* |
| oDT205 | *gccGCTAGCagaaaggaggaggctctgatgagttttttaacggaatgg* |
| oTD241 | *gtgCTCGAGatgcagattgacattgttcaa* |
| oTD261 | *ggcAAGCTTacaaaggatgatggcattgaagcgctttcaatgggtt* |
| oTD263 | *ggcAAGCTTacaaaggatgatggcattgaatgaaatcgctgaggttctc* |
|  |  |
| oDR78 | *gccGGATCCttatttgtatagttcatccatgcc* |
| oDR107 | *ggcAAGCTTacataaggaggaactactatgagtaaaggagaagaac* |
| oDR280 | *gccGAATTCcttcaggttatgaccatctg* |
| oDR281 | *gcggatccCATATGgtctttcggttttctagtgtaacg* |
| oDR344 | *ggcAAGCTTacataaggaggaactactatgggccgctctgagcaaaagc* |
| oDR345 | *cggCTCGAGactattaagatcctcctcgga* |
| oDR409 | *gccGCTAGCtcttcacctgagaaaactgaa* |

| oCM133 | *ccgCTCGAGggttcaaataatgattaaatatc* |
| --- | --- |
| oCM134 | *cgcGCTAGCgccgcgatttccaatgagg* |

capital letters indicate the recognition sites of restriction enzymes
