# Supplementary material for: Novel Secretion Apparatus Maintains Spore Integrity and Developmental Gene Expression in Bacillus subtilis
Source: PLoS Genet. 2009 Jul 17;5(7):e1000566. doi: 10.1371/journal.pgen.1000566 (PMC2703783; doi:10.1371/journal.pgen.1000566)
Supplement: Text S1 — Supplemental materials and methods. (0.06 MB DOC) [file pgen.1000566.s014.doc]

**Supplemental Material and Methods**

***Protein purification and antibody production***

His_6_-SpoIIIAA (full-length), His_6_-SpoIIIAF (an internal fragment) and His_6_-SpoIIIAG (the extracellular domain) fusion proteins were expressed in *E. coli* BL21 DE3 pLysS and purified on Ni^2+^-NTA agarose (Qiagen). Cells were grown in LB at 37˚C to an OD_600_ of 0.8 and induced by addition of IPTG to 1 mM. After 2 h of induction, cells were harvested by centrifugation and resuspended in 1/100^th^ volume buffer I (50 mM Tris-HCl pH 8, 500 mM NaCl, 5 mM 2-Mercaptoethanol, 10 mM imidazole) and flash-frozen in N_2_(l). A crude extract was prepared by freeze-thawing the cells followed by the addition of Lysozyme (1 mg/mL) and sonication. For His_6_-SpoIIIAF and His_6_-SpoIIIAG, a soluble fraction was made by 100,000X g spin and was loaded on a 1 mL Ni^2+^-NTA agarose (Qiagen) column equilibrated with buffer I. Bound protein was washed with buffer I containing 20 mM Imidazole and eluted in buffer I containing 120 mM Imidazole. For His_6_-SpoIIIAA, inclusion bodies were collected after sonication and solubilized for 1 h at 4°C with buffer I containing 6 M Urea. A soluble fraction was made by centrifugation at 20,000X g and the supernatant was loaded on a 1 mL Ni^2+^-NTA agarose (Qiagen) column equilibrated with buffer I containing 6 M Urea. Bound protein was washed with buffer I containing 6 M Urea and 20 mM Imidazole and eluted in buffer I containing 6M Urea and 120 mM Imidazole. Peak fractions were pooled and dialyzed against buffer I containing 1M Urea.

GST-SpoIIIAE (an internal fragment) fusion protein was expressed in *E. coli* NB42 and purified on glutathione-agarose (GE Life Sciences). Cells were grown in LB at 37˚C to an OD_600_ of 0.8 and induced by addition of IPTG to 1 mM. After 2 h of induction, cells were harvested by centrifugation and resuspended in 1/100^th^ volume buffer II (20 mM Tris-HCl pH 8, 500 mM NaCl, 1 mM EDTA, 1 mM EGTA, 5 mM 2-Mercaptoethanol, 1 mM PMSF) and flash-frozen in N_2_(l). A crude extract was prepared by freeze-thawing the cells followed by the addition of Lysozyme (1 mg/mL) and sonication. A soluble fraction was made by 100,000X g spin and was loaded on a 1 mL glutathione-agarose column equilibrated with buffer II. Bound protein was washed with buffer II and eluted in buffer II containing 10 mM glutathione.

***Plasmid construction***

**pDT19** [*amyE::P_spoIIIA-RBSspoIIIAA_-cfp-spoIIIAG (spec)]* was generated in a three-way ligation with a *Hind*III-*Xho*I PCR fragment containing *cfp* with the *spoIIIAA* RBS (oligonucleotide primers oTD3 and oTD4 and template DNA pKM8 [2], an *Xho*I-*Bam*HI PCR fragment containing *spoIIIAG* (oligonucleotide primers oTD6 and oTD7 and wild-type genomic DNA as template) and pDT8 cut with *Hind*III and *Bam*HI. pDT8 [*amyE::P_spoIIIA_ (spec)*] was generated in a two-way ligation with an *Eco*RI-*Hind*III PCR fragment containing P*_spoIIIA_* (oligonucleotide primers oTD1 and oTD2 and wild-type genomic DNA as template) and pDR190 cut with *Eco*RI and *Hind*III. pDR190 [*amyE::spec*] is a derivative of pLD30 [3] that lacks XhoI sites.

**pDT21**  [*spoIIIA^wt^Ω spoIIIAH (phleo)*] was generated in a two-way ligation with a *Hind*III-*Bam*HI PCR fragment containing the last 350 nucleotides of the *spoIIIAH* gene and 50 basepairs downstream of *spoIIIAH* and pDT2 cut with *Hind*III and *Bam*HI. pDT2 [*pUC19-phelo*] was generated in a two-way ligation with an *Eco*RI-*Nde*I PCR fragment containing a phleomycin resistance gene (oligonucleotide primers oDR280 and oDR281 and template DNA pIC22 [4] and pUC19 [5] cut with *Eco*RI and *Nde*I.

**pDT195** [*ycgO::P_spoIIIA-RBSspoIIIAA_-spoIIIAA (erm)*] was generated in a three-way ligation with an *Eco*RI-*Hind*III fragment from pDT15 [2] containing the *spoIIIA* promoter, a *Hind*III-*Bam*HI PCR product containing the *spoIIIAA* gene with the *spoIIIAA* RBS (oligonucleotide primers oDT15 and oTD9 and wild-type genomic DNA as template), and pKM84 [6] cut with *Eco*RI and *Bam*HI.

**pDT196** [*ycgO::P_spoIIIA-RBSspoIIIAA_-spoIIIAB (erm)*] was generated in a three-way ligation with an *Eco*RI-*Nhe*I PCR product containing the *spoIIIA* promoter (oligonucleotide primers oDT1 and oTD200 and wild-type genomic DNA as template), an *Nhe*I-*Bam*HI PCR product containing the *spoIIIAB* gene with *spoIIIAA* RBS (oligonucleotide primers oDT181 and oTD182 and wild-type genomic DNA as template), and pKM84 cut with *Eco*RI and *Bam*HI.

**pDT199** [*ycgO::P_spoIIIA-RBSspoIIIAA_-spoIIIAE (erm)*] was generated in a three-way ligation with an *Eco*RI-*Hind*III fragment from pDT15 containing the *spoIIIA* promoter, an *Hind*III-*Bam*HI PCR product containing the *spoIIIAE* gene with *spoIIIAA* RBS (oligonucleotide primers oDT190 and oTD191 and wild-type genomic DNA as template), and pKM84 cut with *Eco*RI and *Bam*HI.

**pDT201** [*ycgO::P_spoIIIA-RBSspoIIIAA_-spoIIIAG (erm)*] was created in a three-way ligation with an *Eco*RI-*Hind*III fragment from pDT15 containing the *spoIIIA* promoter, an *Hind*III-*Bam*HI PCR product containing the *spoIIIAG* gene with *spoIIIAA* RBS (oligonucleotide primers oDT196 and oTD7 and wild-type genomic DNA as template), and pKM84 cut with *Eco*RI and *Bam*HI.

**pDT202** [*ycgO::P_spoIIIA-RBSspoIIIAA_-spoIIIAH (erm)*] was created in a three-way ligation with an *Eco*RI-*Hind*III fragment from pDT15 containing the *spoIIIA* promoter, an *Hind*III-*Bam*HI PCR product containing the *spoIIIAH* gene with *spoIIIAA* RBS (oligonucleotide primers oDT198 and oTD11 and wild-type genomic DNA as template), and pKM84 cut with *Eco*RI and *Bam*HI.

**pDT204** [*ycgO::P_spoIIIA-RBSspoIIIAA_-spoIIIACD (erm)*] was generated in a three-way ligation with an *Eco*RI-*Hind*III fragment from pDT15 containing the *spoIIIA* promoter, an *Hind*III-*Bam*HI PCR product containing the *spoIIIAC* and *spoIIIAD* genes with *spoIIIAA* RBS (oligonucleotide primers oDT184 and oTD188 and wild-type genomic DNA as template), and pKM84 cut with *Eco*RI and *Bam*HI.

**pDT246** [*amyE::P_spoIIIA-RBSspoIIIAA_-myc3-spoIIIAD (spec)*] was created in a two-way ligation with an *XhoI*-*Bam*HI PCR product containing the *spoIIIAD* gene with *spoIIIAA* RBS (oligonucleotide primers oTD241 and oTD188 and wild-type genomic DNA as template) and pDT62 cut with *Xho*I and *Bam*HI. pDT62 [*amyE::P_spoIIIA-RBSIIIA_-myc3-spoIIIAG (spec)*] was generated in a two-way ligation with an *Hind*III*-Xho*I PCR product containing a *myc*3 repeat with an optimized RBS (oligonucleotide primers oDR344 and oDR345 and pKL94 [7] and pDT19 cut with *Hind*III and *Xho*I.

**pDT257** [*amyE::P_spoIIIA-RBSIIIAA_-spoIIIAA^D224A^ (cat)*] was generated in a three-way ligation with an *Eco*RI-*Hind*III fragment from pDT15 containing the *spoIIIA* promoter, a PCR-amplified fragment containing *spoIIIAA^D224A^* and *spoIIIAA* RBS (oligonucleotide primers oDT9 and oTD15 and plasmid pDT144 as template) and pDG1662 [8] cut with *Eco*RI and *Bam*HI. pDT144 [*amyE::P_spoIIIA-RBSspoIIIAA_-cfp-spoIIIAA^D224A^ (spec)*] was built by site-directed mutagenesis using oligonucleotide primers oTD135 and oTD136 and template DNA pDT17. pDT17 [*amyE::P_spoIIIA-RBSspoIIIAA_-cfp-spoIIIAA^wt^ (spec)*] was built in a three-way ligation with a *Hind*III-*Xho*I PCR fragment containing *cfp* with *spoIIIAA* RBS (with *B. subtilis* codons) (oligonucleotide primers oTD3 and oTD4 and template DNA pKM8), an *Xho*I-*Bam*HI PCR fragment containing *spoIIIAA* (oligonucleotide primers oTD8 and oTD9 and wild-type genomic DNA as template) and pDT8 cut with *Hind*III and *Bam*HI.

**pDT259** [*yycR::P_sspE_-cfp (cat)*] was generated in a two-way ligation with an *Eco*RI-*Bam*HI fragment from pDT244 containing *PsspE-cfp* and pNS037 cut with *Eco*RI and *Bam*HI. pNS037 is an ectopic integration vector for double crossover insertions into the nonessential *yycR* chromosomal locus with the chloramphenicol resistance gene (N. Sullivan and D.Z.R., unpublished).

**pDT266** [*amyE::P_spoIIIA-RBSIIIAA_-spoIIIAA^wt^ (spec)*] was generated in a two-way ligation with a PCR amplified fragment containing *spoIIIAA* and *spoIIIAA* RBS (oligonucleotide primers oDT9 and oTD15 and plasmid pDT17 as template) and pDT8 cut with *Hind*III and *Bam*HI.

**pDT267** [*amyE::P_spoIIIA-RBSIIIAA_-spoIIIAA^D224A^ (spec)*] was generated in a two-way ligation with a PCR amplified fragment containing *spoIIIAA*^D224A^ and *spoIIIAA* RBS (oligonucleotide primers oDT9 and oTD15 and plasmid pDT144 as template) and pDT8 cut with *Hind*III and *Bam*HI.

**pDT307** [*ycgO::P_sspE-RBSopt_-gfp (spec)*] was generated in a three-way ligation with an *Eco*RI-*Hind*III fragment containing *PsspE* (retrieved from pDT259) and a *Hind*III-*Bam*HI fragment containing the *gfp* gene and an optimized RBS (retrieved from pDT235), and pKM83 cut with *Eco*RI and *Bam*HI. pKM83 [*ycgO::spec*] is an ectopic integration vector for double crossover insertions into the nonessential *ycgO* chromosomal locus with the spectinomycine resistance gene (K. Marquis and D.Z.R., unpublished). pDT235 [*ycgO::P_spoIIQ-RBSopt_-gfp (erm)*] was built in a two-way ligation with a PCR amplified fragment containing the *gfp* gene and an optimized RBS (oligonucleotide primers oDR107 and oDR78 and plasmid pKL147 [9] as template) and pDT79 [6] cut with *Hind*III and *Bam*HI.

**pDT326** [*ycgO::P_spoIIIA-RBSspoIVFA_-ttg-spoIIIAE (erm)*] was created in a two-way ligation with a *Hind*III-*Bam*HI PCR fragment containing *spoIIIAE* with *spoIVFA* RBS and a TTG start codon (oligonucleotide primers oTD261 and oTD191 and wild-type genomic DNA as template) and pDT199 (this study) cut with *Hind*III and *Bam*HI.

**pDT330** [*ycgO::P_spoIIIA-RBSspoIVFA_-ttg-spoIIIAA (erm)*] was generated in a two-way ligation with a *Hind*III-*Bam*HI PCR fragment containing *spoIIIAA* with *spoIVFA* RBS and a TTG start codon (oligonucleotide primers oTD263 and oTD9 and wild-type genomic DNA as template) and pDT199 cut with *Hind*III and *Bam*HI.

**pCM16** [*ycgO::P_spoIIIA-RBSspoIIIAA_-spoIIIAF (erm)*] was generated in a two-way ligation with an *Nhe*I-*Bam*HI PCR product containing the *spoIIIAF* gene with *spoIIIAA* RBS (oligonucleotide primers oDT205 and oTD201 and wild-type genomic DNA as template) and pDT196 cut with *Nhe*I and *Bam*HI.

**pCM161** [*lacA::P_spoIIQ_-sigG (tet)*] was generated in a two-way ligation with an *Xho*I-*Bam*HI PCR product containing *P_spoIIQ_-sigG* (oligonucleotide primers oCM133 and oCM134 and genomic DNA from strain MO03196 [10]) and pNC18 cut with *Xho*I and *Bam*HI. pNC18 [*lacA::tet*] is an ectopic integration vector for double crossover insertions into the nonessential *lacA* locus (N. Campo and DZR unpublished). The ligation product was transformed directly into *B. subtilis*.

**pCM162** [*yhdG::P_spoIIQ_-sigG (erm)*] was generated in a two-way ligation with an *Xho*I-*Bam*HI PCR product containing *P_spoIIQ_-sigG* (oligonucleotide primers oCM133 and oCM134 and genomic DNA from strain MO03196) and pBB279 cut with *Xho*I and *Bam*HI. pBB279 [*yhdG::erm*] is an ectopic integration vector for double crossover insertions into the nonessential *yhdG* locus (B. Burton and DZR unpublished). The ligation product was transformed directly into *B. subtilis*.

**pNS38** [*yvbJ::P_spoIIQ_-cfp (spec)*] was generated in a two-way ligation with an EcoRI/BamHI fragment containing *P_spoIIQ_-cfp* and obtained from pKM8 and pNS28 cut with *Eco*RI and *Bam*HI. pNS28 [*yvbJ::spec*] is an ectopic integration vector for double crossover insertions into the nonessential *yvbJ* locus (N. Sullivan and DZR unpublished).

**pDT152** [*his_6_-spoIIIAA*] was generated in a two-way ligation with a *Nhe*I-*Xho*I PCR product containing the *spoIIIAA* gene (oligonucleotide primers oTD150 and oTD151 and template DNA pDT17) and pRsetA (Invitrogen) cut with *Nhe*I and *Xho*I.

**pDT170** [*his_6_-spoIIIAF_(fragment)_*] was generated in a two-way ligation with a *Nhe*I-*Xho*I PCR product encoding an internal SpoIIIAF fragment (152 amino acids) (oligonucleotide primers oTD169 and oTD170 and wild-type genomic DNA as template) and pRsetA cut with *Nhe*I and *Xho*I.

**pDT178** [*GST-spoIIIAE_(fragment)_*] was created in a two-way ligation with a *Bam*HI-*Xho*I PCR product encoding an N-terminal fragment of SpoIIIAE (85 amino acids) (oligonucleotide primers oTD176 and oTD164 and wild-type genomic DNA as template) and pGEX-6P-2 (GE Life Sciences) cut with *Bam*HI and *Xho*I.

**pKM108** [*his_6_-spoIIIAG_(fragment)_*] was generated in a two-way ligation with a *Nhe*I-*Bam*HI PCR product encoding SpoIIIAG extracellular domain (179 amino acids) (oligonucleotide primers oDR409 and oTD7 and wild-type genomic DNA as template) and pRsetA cut with *Nhe*I and *Bam*HI.

**Supplemental References**

1. Sharp MD, Pogliano K (1999) An in vivo membrane fusion assay implicates SpoIIIE in the final stages of engulfment during Bacillus subtilis sporulation. Proc Natl Acad Sci U S A 96: 14553-14558.

2. Doan T, Marquis KA, Rudner DZ (2005) Subcellular localization of a sporulation membrane protein is achieved through a network of interactions along and across the septum. Mol Microbiol 55: 1767-1781.

3. Garsin DA, Paskowitz DM, Duncan L, Losick R (1998) Evidence for common sites of contact between the antisigma factor SpoIIAB and its partners SpoIIAA and the developmental transcription factor sigmaF in Bacillus subtilis. J Mol Biol 284: 557-568.

4. Steinmetz M, Richter R (1994) Plasmids designed to alter the antibiotic resistance expressed by insertion mutations in Bacillus subtilis, through in vivo recombination. Gene 142: 79-83.

5. Yanisch-Perron C, Vieira J, Messing J (1985) Improved M13 phage cloning vectors and host strains: nucleotide sequences of the M13mp18 and pUC19 vectors. Gene 33: 103-119.

6. Doan T, Rudner DZ (2007) Perturbations to engulfment trigger a degradative response that prevents cell-cell signalling during sporulation in Bacillus subtilis. Mol Microbiol 64: 500-511.

7. Perez AR, Abanes-De Mello A, Pogliano K (2000) SpoIIB localizes to active sites of septal biogenesis and spatially regulates septal thinning during engulfment in bacillus subtilis. J Bacteriol 182: 1096-1108.

8. Guerout-Fleury AM, Frandsen N, Stragier P (1996) Plasmids for ectopic integration in Bacillus subtilis. Gene 180: 57-61.

9. Lemon KP, Grossman AD (1998) Localization of bacterial DNA polymerase: evidence for a factory model of replication. Science 282: 1516-1519.

10. Karmazyn-Campelli C, Rhayat L, Carballido-Lopez R, Duperrier S, Frandsen N, et al. (2008) How the early sporulation sigma factor sigmaF delays the switch to late development in Bacillus subtilis. Mol Microbiol 67: 1169-1180.
